# Employing fingerprinting of medicinal plants by means of LC-MS and machine learning for species identification task

**DOI:** 10.1038/s41598-018-35399-z

**Published:** 2018-11-19

**Authors:** Pavel Kharyuk, Dmitry Nazarenko, Ivan Oseledets, Igor Rodin, Oleg Shpigun, Andrey Tsitsilin, Mikhail Lavrentyev

**Affiliations:** 1 Skolkovo Institute of Science and Technology, Center for Computational and Data-Intensive Science and Engineering, Moscow, 143026 Russia; 2 Institute of Numerical Mathematics of the Russian Academy of Sciences, Moscow, 119991 Russia; 3 Lomonosov Moscow State University, Faculty of Chemistry, Moscow, 119991 Russia; 4 All-Russian Research Institute of Medicinal and Aromatic Plants (VILAR), Moscow, 117216 Russia; 5 Saratov State University, Department of Botanics and Ecology, Saratov, 410012 Russia

## Abstract

A dataset of liquid chromatography-mass spectrometry measurements of medicinal plant extracts from 74 species was generated and used for training and validating plant species identification algorithms. Various strategies for data handling and feature space extraction were tested. Constrained Tucker decomposition, large-scale (more than 1500 variables) discrete Bayesian Networks and autoencoder based dimensionality reduction coupled with continuous Bayes classifier and logistic regression were optimized to achieve the best accuracy. Even with elimination of all retention time values accuracies of up to 96% and 92% were achieved on validation set for plant species and plant organ identification respectively. Benefits and drawbacks of used algortihms were discussed. Preliminary test showed that developed approaches exhibit tolerance to changes in data created by using different extraction methods and/or equipment. Dataset with more than 2200 chromatograms was published in an open repository.

## Introduction

Analytical chemistry of medicinal plants is experiencing continuous expansion in the last decades^1–3^. Complex samples with no obvious targets for identification and quantitation have given rise to widespread use of multivariate statistics and data mining approaches^4^. This especially applies to China’s pharmacology, which strives to upgrade its Traditional Chinese Medicine (TCM) practices (herbal medicine included) up to the modern clinical and pharmacological standards^5–7^. Naturally, for a herbal medicine to be recognized as certified drug, multiple clinical studies are required to determine its efficiency and safety.

This goal meets at least two major problems when faced with plant extracts, namely standardization of herbal drugs and interpretation of clinical studies results. As for mechanisms of action and interpretation of treatment results in clinical studies, complex plant extracts and their mixtures may contain up to hundreds or more physiologically active compounds, which makes thorough interpretation nigh unreachable, at least for now. The former one is rather self-evident-lack of standardization naturally leads to further problems in quality control during production steps^8–10^. This complication may be addressed by established pharmacological approaches based on individual standards for each active compound in a drug, but such analysis would be economically and practically unfeasible.

Profiling or fingerprinting emerged as a powerful alternative to classical analytical methodology^11–13^. In profiling it is assumed that raw analytical data includes information sufficient to answer biological question at hand. Therefore, the task is to use some approach to find that useful information and separate it from noise. This is usually done on a dataset with samples from various states to be distinguished between such as authentic/counterfeit^14^, pure/adulterated^12,15^, distinguishing plant species^16^, geographical origins^17,18^ and other similar cases^19–21^. By applying various techniques it is possible to reliably extract variables that allow to discriminate between above-mentioned states. This classification task can be done by various means: artificial neural networks (ANN), projection to latent structures discriminant analysis (PLS-DA), support vector machine (SVM) and many others, which were extensively discussed in review by Ning *et al*.^22^. Many of this techniques belong to the field of machine learning (ML), science about algorithms which can learn from data and make predictions. Nevertheless, despite being very powerful in many aspects, combination of profiling and ML has its own drawbacks and limitations. More often than not, ML methods operate on “black box” principle, i.e. one cannot easily interpret on what basis a trained algorithm makes its decisions^23^. In other words, it may be hard to map strongly weighted components of its structure to actual properties of objects or phenomena. Probabilistic graphical models (PGM) such as Bayesian Networks (BN) may be an alternative in this case^24^. PGMs are widely used in machine learning to solve classification tasks from the wide range of scientific and industrial fields, including analytical chemistry^25–27^. One of their advantages is clear visualization of dependencies between variables, which can potentially help to understand classification criteria of an algorithm and map them back to properties of objects in question. Moreover, Bayesian Networks belong to generative models and are capable of producing artificial data^28^, which can help to compensate for small datasets.

One of the key problems in implementing efficient classification algorithm is the choice of feature space. Single liquid chromatography–mass-spectrometry (LC-MS) sample contains millions of data points (raw data) or hundreds of chromatographic peaks (after integration and peak extraction). Faced with strictly limited size of available data, it is also imperative to try to find the smallest possible set of variables in data which can still result in maximum classification accuracy of the final algorithm. An appealing way in this situation is to use autoencoder or tensor decompositions as feature extractors. Autoencoder is a type of artificial neural network (ANN) which may be used for dimensionality reduction of input data^29,30^. Unlike commonly used principal component analysis (PCA), which only seeks axes of biggest variance, autoencoder is forced to reconstruct full input data with minimal loss. That is done by capturing and utilizing internal structure of data. The output of the encoding part of autoencoder with the greatly reduced number of variables can subsequently be used in ML model for training classification algorithm. Similarly, tensor decompositions can be used to map data in low dimensional spaces and to separate variables.

The other important problem in regard to medicinal plants analysis is that it may be hard to decide criteria for samples to be assigned class labels in the first place. General plant identification algorithm, capable of recognizing plant species with data from chemical analysis could be a good starting point in this regard. Although there were some steps in this direction in various forms^31–33^, no finalized algorithm had been created. Earlier we preliminarily confirmed on a small scale, that it is possible to get classification accuracy of about 95% for medicinal plant identification task^34^ by using the data from plant extracts analyzed by LC-MS. Common machine learning techniques like logistic regression, SVM and random forest were used to directly map feature space into class tags. This work is an attempt to further enhance the proposed approach with use of Bayesian Networks, Tucker decomposition and autoencoder neural network for more in-depth analysis of LC-MS data and its application for plant species identification. This work is based on a dataset of morphologically identified plants and commercial plant material. We also tried to test practical applicability of trained algorithms when using data obtained with extraction protocols and LC-MS platforms different from that in the main dataset.

## Results

Cumulatively, about 2200 chromatograms were generated from medicinal plant extracts of 74 species, which are listed in Table 2. The scientific names of the plants are given according to The Plant List^35^. Small set of chromatograms (9 species) obtained using alternative extraction procedures and/or LC-MS platform was used for additional testing (Test 2). In the choice of experimental material, almost all the species covered by Russian Pharmacopoeia (about 50) along with some local medicinal plant species were selected. 18 of the species with less than 20 chromatograms were united into a separate negative class (about 10% of dataset) to get more robust classification results. Negative class was designed for the “winner takes all” strategy where algorithms only present one answer with the highest score. Its intended purpose was for an algorithm to assign “negative class label to samples of species which are not presented in data bank. Composition of the rest of the dataset is summarized in Supplementary Fig. S1.6.

**Figure 1:**
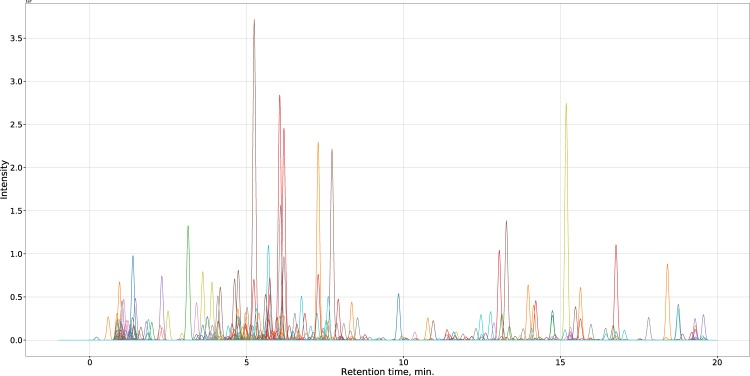
Synthetic chromatrogram reconstructed from one of the sample vectors for *Anethum graveolens* in the dataset. Values from all 1600 variables (*m*/*z* values 100–900, negative and positive polarities) were simulated as Gaussian peaks. Area of each individual peak is directly proportional to magnitude of corresponding value in data vector. Retention times were retrieved from the original LC-MS data. Peak colors were chosen randomly.

Keeping in mind actual applicability, some variables highly dependent on the configuration of LC-MS platform were discarded. As scalability (larger species pool) is also an important factor, all choices like a limited set of chromatographic peaks also could not be used as a feature space. Similarly, retention times of compounds, which change according to the type of stationary and mobile phases, gradient program etc. are also not a feasible choice. As such, a vector of 1600 variables containing only peak areas for a range of *m*/*z* values was generated for each chromatogram. Example of chromatogram reconstructed from such vector can be seen in Fig. 1.

As a first choice for classification algorithm, Bayesian Networks (BN) were selected. Values for 30% peaks with highest abundances were set to 1 and the rest of the values were set to 0. Such layout was used to investigate whether plant species could be distinguished simply based on presence or absence of peaks with certain *m*/*z* values, i.e. purely qualitative approach. Discretized BN learned from the dataset had resulted in classification accuracy of 90% on Test 1 (Table 1 Part 1).

**Table 1:** Comparative characteristics of implemented approaches. Test 2 is independent from Train/Test 1 parts. In Part 1 and Part 3 all values presented are medians across 5-times repeated 5-fold cross validation runs. In Part 2 the same partitioning was used but final results were computed as top-N’s (see Supplementary S1.2).

Part 1. Results for “winner takes all” strategy. Prediction times are written per one sample. For classifiers based on features spaces learned with autoencoder additional times for estimation of autoencoder parameters are given in parentheses										
Method	Accuracy, %			F1, %			Time			
	Train	Test 1	Test 2	Train	Test 1	Test 2	Training	Prediction		
Logistic regression (autoencoded)	99.7	96.5	72.7	99.7	96.4	77.3	1 m 16 s (+1 h 30 m)	0.06 ms		
Naive Bayes (autoencoded)	89.6	84.5	77.3	89.8	84.6	83.3	8 ms (+1 h 30 m)	0.02 ms		
Hybrid BN (autoencoded)	92.2	87.2	68.2	92.4	87.1	74.8	50 m 47 s (+1 h 30 m)	1.8 ms		
Large discrete BN	—	90.0	72.7	—	90.0	81.0	3 m 14 s	9 m		
Sparse NTD (principal angle)	97.6	93.4	86.4	97.6	93.3	91.1	18 h 19 m	1.1 s		
Sparse NMF (principal angle)	99.2	94.8	81.8	99.2	94.9	84.1	28 m 46 s	1.1 s		
**Part 2. TopN approach. Output is considered to be accurate when correct label is present in TopN results**.										
**Method**	**Accuracy, %**									
	**Test 1**					**Test 2**				
	**Top1**	**Top2**	**Top3**	**Top4**	**Top5**	**Top1**	**Top2**	**Top3**	**Top4**	**Top5**
Logistic regression (autoencoded)	96.5	98.5	99.1	99.3	99.5	72.7	79.6	84.1	84.1	86.4
Naive Bayes (autoencoded)	84.5	91.6	94.2	95.7	96.7	77.3	86.4	88.6	93.2	93.2
Large discrete BN	90.0	93.8	95.1	95.1	95.3	72.7	81.8	88.6	90.9	93.2
Sparse NTD (principal angle)	93.4	95.9	96.6	97.1	97.4	86.4	88.6	90.9	90.9	93.2
Sparse NMF (principal angle)	94.8	96.2	96.5	96.9	97.1	81.8	84.1	86.4	86.4	88.6
**Part 3. Plant organ identification**.										
**Method**	**Accuracy, %**			**F1, %**						
	**Train**	**Test 1**	**Test 2**	**Train**	**Test 1**	**Test 2**				
Logistic regression (autoencoded)	86.3	83.1	68.2	86.1	82.6	64.1				
Naive Bayes (autoencoded)	76.6	74.7	63.6	76.1	74.2	58.3				
Hybrid BN (autoencoded)	76.4	74.7	65.9	76.1	73.9	63.0				
Sparse NTD (principal angle)	89.9	87.6	86.4	90.3	87.9	87.7				
Sparse NMF (principal angle)	96.2	94.2	84.1	96.3	94.3	84.6

**Table 2:** Plant species used in experiment.

Part of the plant	Plant species
Roots or rhizomes	*Eleutherococcus sessiliflorus (Rupr. & Maxim.) S.Y.Hu*, *Eleutherococcus senticosus (Rupr. & Maxim.) Maxim*., *Oplopanax elatus (Nakai) Nakai*, *Panax ginseng C.A.Mey*., *Rhodiola rosea L*., *Inula helenium L*., *Helianthus tuberosus L*., *Angelica archangelica L*., *Acorus calamus L*., *Rosa majalis Herrm*., *Valeriana officinalis L*., *Sambucus nigra L*., *Glycyrrhiza glabra L*., *Levisticum officinale W.D.J.Koch*, *Cichorium intybus L*., *Arctium lappa L*., *Potentilla erecta (L.) Raeusch*., *Dioscorea caucasica Lipsky*, *Taraxacum officinale (L.) Weber ex F.H.Wigg*., *Hedysarum neglectum Ledeb*., *Aralia elata var. mandshurica (Rupr. & Maxim.) J.Wen*, *Astragalus membranaceus (Fisch.) Bunge*, *Bergenia crassifolia (L.) Fritsch*, *Polemonium caeruleum L*., *Althaea officinalis L*.
Seeds or fruit	*Coriandrum sativum L*., *Daucus carota L*., *Petroselinum crispum (Mill.) Fuss*, *Foeniculum vulgare Mill*., *Anethum graveolens L*., *Pimpinella anisum L*., *Silybum marianum (L.) Gaertn*., *Linum usitatissimum L*., *Aronia melanocarpa (Michx.) Elliott*, *Rhamnus cathartica L*., *Juniperus communis L*., *Prunus padus L*., *Vaccinium myrtillus L*., *Humulus lupulus L*.
Leaves or flowers or aboveground part	*Bupleurum aureum Fisch. ex Hoffm*., *Pimpinella saxifraga L*., *Heracleum sphondylium subsp. sibiricum (L.) Simonk*., *Asarum europaeum L*., *Aegopodium podagraria L*., *Betula pendula Roth*, *Sambucus nigra L*., *Ginkgo biloba L*., *Melilotus officinalis (L.) Pall*., *Origanum vulgare L*., *Fragaria vesca L*., *Hypericum perforatum L*., *Viburnum opulus L*., *Urtica dioica L*., *Frangula alnus Mill*., *Tilia cordata Mill*., *Tussilago farfara L*., *Mentha* × *piperita L*., *Calendula officinalis L*., *Tanacetum vulgare L*., *Plantago major L*., *Artemisia absinthium L*., *Leonurus quinquelobatus Gilib*., *Matricaria chamomilla L*., *Senna alexandrina Mill*., *Pinus sylvestris L*., *Populus balsamifera L*., *Viola tricolor L*., *Equisetum arvense L*., *Thymus serpyllum L*., *Salvia officinalis L*., *Aerva lanata (L.) Juss*., *Echinacea purpurea (L.) Moench*, *Bidens tripartita L*., *Convallaria keiskei Miq*., *Helichrysum arenarium (L.) Moench*

It is safe to say that 1600 variables is too much, both in regard to the dataset size and computational costs, especially considering the absence of desired classification accuracy. Reasonable reductions of feature space can also speed up any computational algorithm. For this purpose autoencoder was selected. Encoded data vectors with 25 variables were used to train logistic regression and continuous Bayes classifiers (both Naive Bayes and hybrid Bayesian Network) with resulting identification accuracy of 96% and 84–87% on Test 1 respectively. All above-mentioned models showed accuracy of 68–77% on Test 2.

**Figure 2:**
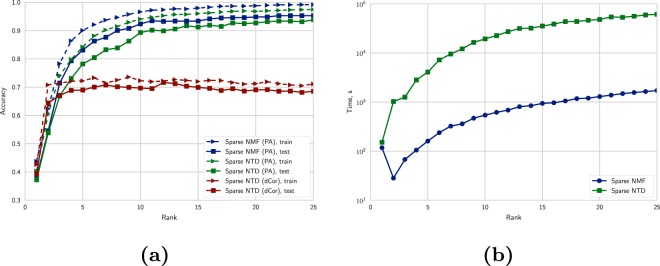
Rank selection for sparse NMF/NTD: (**a**) comparative plot of accuracies for two metrics, principal angle and distance correlation as base of classifying rule for Tucker decomposition, and sparse NMF with principal angle; (**b**) time required to estimate factor matrices. In both subfigures medians across 5-fold cross-validation runs are presented.

An alternative to the autoencoder was to separate dimensions of data with Tucker decomposition, revealing multilinear dependencies between them. Non-negative and sparsity constraints were applied to parameters of the decomposition. Factor matrices of two axes (m/z and polarity) were used for further classification as described in “Methods” section. There are two important points to note here: rank selection and selection of distance measure between column-spaces (linear span of column-vectors) of factor-matrix (Fig. 2(a)). Comparing distance metrics, principal angle vividly outperformed distance correlation on higher Tucker ranks of m/z mode, thus further experiments were performed with only this metric. However, higher rank values imply longer computations (Fig. 2(b)).

**Figure 3:**
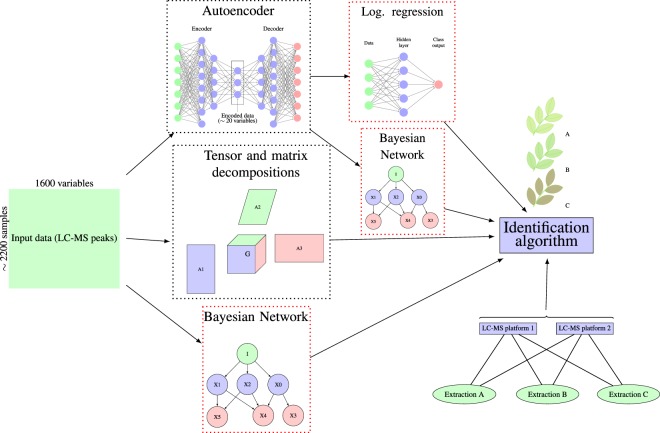
Schematic representation of all computational experiments conducted in this study. Dataset was utilized in 3 alternative ways: steep dimensionality reduction through autoencoder followed by either logistic regression or Bayesian classifiers, constrained matrix and Tucker decompositions with classifier, and direct application of discrete Bayesian Network on data. All classification algorithms were tested with 5-times repeated 5-fold cross-validation.

One of advantages of this approach is that adding new classes does not involve re-estimation of already computed factor matrices. Accuracy values for Tucker rank of m/z axis equal to 25 being high enough and the gap between training and validation accuracy curves being sufficiently small were the reasons it was chosen for cross-validated comparison with other methods. According to the Table 1 Part 1, classifier based on Tucker decomposition with principal angle distance measure performs well (93% and 86% respectively for Test 1 and Test 2). Although it has larger gap between performances on Train and Test 1 parts in comparison to logistic regression on autoencoded data, at the same time it shows the best results on independent data (Test 2) classification. Matrix factorization with the same constraints was also implemented as a reference point. In this case, instead of representing dataset as 3D array with axes *sample, m/z and polarity*, two last axes were unfolded. General outline of this study is summarized in Fig. 3 and performance of all implemented algorithms in Table 1 Part 1.

## Discussion

Various approaches were tested to build efficient and robust plant species identification algorithm. Results show that with careful selection of feature space and model tuning it is possible to achieve up to 96% classification accuracy even with large and heterogeneous negative class. For more in-depth analysis of performance, confusion matrices for each algorithm were examined (Supplementary Fig. S1.1). Confusion matrices show what labels were assigned to samples and how often, helping monitor various internal problems. Most misclassification cases were accounted for by samples being mistakenly put in the negative class or vice versa, i.e. when in doubt, algorithms tended to put samples in the negative class rather than assign it some other tag, but some negative class samples also were mislabeled. Notable exceptions are pairs *Bidens Tripartita* – *Anethum graveolens* and *Aerva lanata* – *Salvia officinalis*, which were consistently mistaken for each other (up to ~30% for some algorithms). This can be attributed to similarities in data vectors. It was also shown that models learned on data obtained in a single set of conditions (LC-MS platform and extraction procedure) can be used to identify samples from different sources with reasonable accuracy. In that regard, the best performance was shown by combination of Tucker decomposition and Principle Angle measurements with Logistic Regression performing significantly worse, showing signs of overfitting.

**Figure 4:**
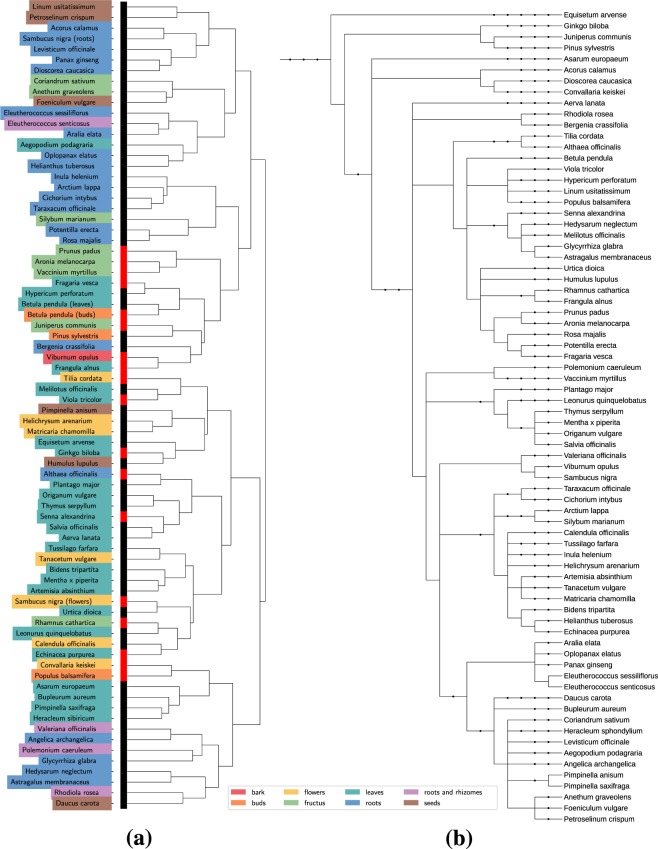
Clustering analysis. (**a**) HCA dendrogram for mean sample vectors of 76 classes used in the study. Red markers show species pooled to form negative class. (**b**) Phylogenetic tree of the corresponding 74 species set. Dots represent classification units: genera, families etc. Drawn based on NCBI taxonomy with the help of PhyloT platfrom^36^.

To represent relative positions of the selected species in respect to each other, phylogenetic tree (Fig. 4(b)) was constructed with the help of PhyloT platform^36^. Then, Hierarchical Clustering Analysis (HCA) was employed to explore similarities between actual phylogenetic relationships and groupings caused by the dataset’s internal structure (Fig. 4(a)). In conditions where chemical data from various plant parts (roots, leaves, flowers etc.) was used as base data for distance measuring, it is natural to get limited results from clustering analysis. Across many linkage and distance metrics tested, HCA generally tended to correctly group some of the closely (same genus or family) related species if the same plant parts were used and failed to form adequate groupings of higher orders. This was true for both raw and encoded data vectors. HCA was also computed for 2200 samples (Supplementary Fig. S1.3) and it showed some impovement in the sense of distances between samples from the same class being smaller after encoding. Correspondingly, visualization with t-SNE (Supplementary Fig. S1.5) showed mostly minor improvements in data structure after encoding.

**Figure 5:**
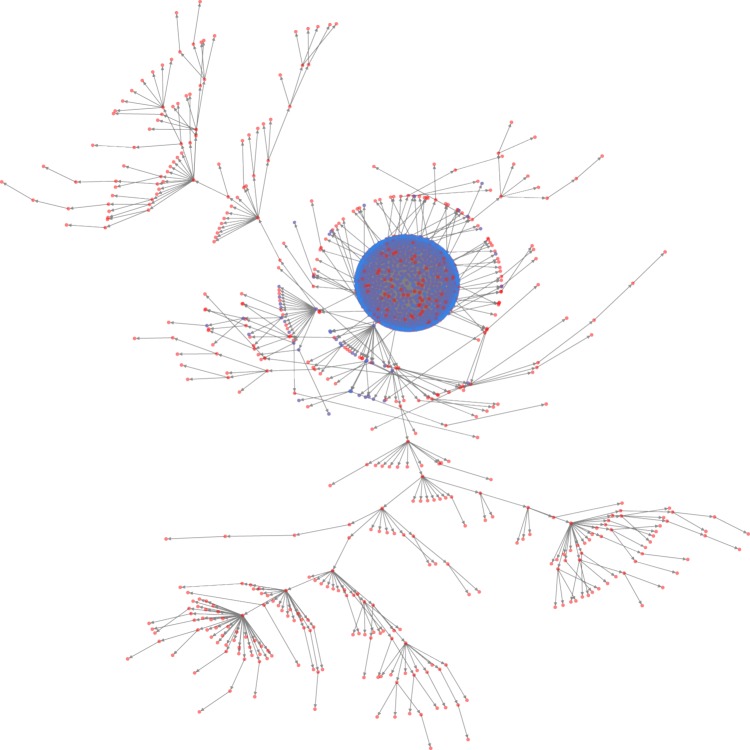
Example of discrete BN structure learned from 1600 variables. Dense variable cluster is centered around “identity” variable. This variable with 59 possible states was responsible for class prediction. All other nodes represent *m*/*z* values of chromatographic peaks with edges representing conditional dependency between variables. Blue nodes highlight structure motif identical for each network learned in CV. This network was also saved in.sif format (can be found in our repository by the link given below).

Despite being generated on LC-MS instrumentation, our data preprocessing left mostly mass-spectrometry related data – *m*/*z* and peak area values for detected compounds. Naturally, across domain of higher plants, each m/z value rounded to integer format can represent from a few and up to tens of compounds, which would lack both function and structure similarity. Thus, results of HCA on 76 classes was to be expected. Example of structure of a learned Bayesian Network with 1600 variables (Fig. 5) can be helpful in facilitating this point. Nodes involved in complex multilayered conditional dependencies and consistent during cross-validation were present in a very limited number (~20). Majority of the variables were learned as being directly dependent on the class variable, i.e. belonging to Naive Bayes type of classification algorithms. If, for example, nodes were represented by a set of specific secondary metabolites, one would expect to find more meaningful and complex inter-dependencies in the structure of a learned network. Thus, distances between data vectors produced by following the proposed protocol, do not necessarily correlate with underlying phylogenetic relationships of species involved.

A bit differently in this aspect situation was with SNMF and SNTD. 3 rows (factors) from factor matrices with highest intra-species and lowest inter-species correlations were selected for MF and TD (Supplementary Fig. S1.4). Due to factor matrices being computed individually for each species, they contain information about characteristic sets of compounds with corresponding relative abundances for a particular species. Both tensor and matrix decomposition techniques prediction results on Test 1 (similar to train set) were highly accurate, while on more heterogeneous Test 2 set TD noticeably outperformed matrix factorization. It’s likely that while SNMF has twice as much variables in each factor compared to TD and thus slightly better captures train data and similar Test 1, it loses to TD in terms of generalization.

By making algorithms show more candidate classes (Table 1 Part 2), performance of computed models rises significantly. It is more apparent in case of Test 2 dataset, which contained data from samples with alternative extraction procedures or/and acquired on a different instrumentation.

The most obvious increase was shown by large BN on Test 2, where emergence of correct labels in Top5 jumped by more than 20% compared to “winner takes all” approach. Although exact accuracy values may drop when using larger and more diverse datasets, this shows great potential of discrete BNs in such applications. All in all, TopN representation can be considered a more preferable way of output – narrowing possible candidates to 3–5 with ~95% or more accuracy can be more beneficial than 80% accurate single candidate species.

**Figure 6:**
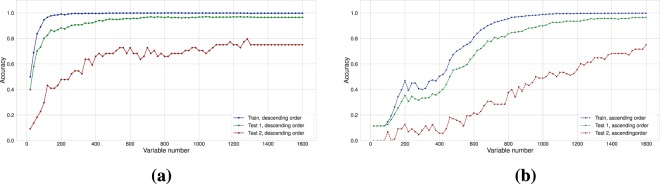
Importance analysis of variables in the data vectors. (**a**) Accuracy of Logistic Regression learned on step-wise increasing variable set. Variables were sorted using weights from 1st layer of autoencoder in descending order. (**b**) The same principle, only variables were sorted using weights in ascending order.

“Neighbor analysis” was also implemented (highlighting most frequent hits emerging in TOP5 results for a particular species) – it was used to monitor which samples are considered to be similar by the algorithms (Supplementary Fig. S1.2). Examining top neighbors did not elicit notable correlations between phylogenetic inter-species distances and frequency of species being mutually in Top5 recognition results for each other. To evaluate which of 1600 features contributed to discriminating power of our dataset the most, weight matrices from autoencoder were used as a tentative marker for importance of variables. All variables were sorted according to norms of 1st layer weight matrix and used in two ways: direct and reverse. Accuracy of LR learned on a step-wise increasing direct variable set (Fig. 6(a)) shows that it is hard to discard more than half of the variables without significant loss of performance. At the same time, Fig. 6(b) where reverse order was used, shows considerably lower efficiency of variables with low corresponding weights. Although it is safe to assume that higher weighted variables represent compounds with higher discriminating power, exact lists of such compounds strongly depend on initial dataset composition. In that case, instead of discarding a few hundred variables, encoding entire LC-MS run worth of data into 25 variables (via autoencoder) seems to be more beneficial as it makes visualization and learning process for any algorithm much faster.

Strictly speaking, each of the 76 classes correspond not to a species but a pair (species, organ), e.g. *Sambucus Nigra* is represented by two classes: (*Sambucus Nigra*, roots) and (*Sambucus Nigra*, flowers). By using organ information for all samples, feasibility of plant organ identification was also examined (Table 1 Part 3). Altogether, 7 classes were formed: bark, buds, flowers, fructus, roots and rhizomes, roots and seeds. Algorithms showed high distinguishing ability between most classes (up to 92% accuracy), excluding very similar pair of classes (roots, roots and rhizomes). Even though different plant organs exhibit pronouncedly different metabolism and therefore chemical composition, it remains inconclusive whether they can be efficiently identified on large scale with proposed setting. Primary reason for such consideration is the nature of examined dataset: considering high identification accuracy of (species, organ)-classes, their combinations (i.e. organ classes) may also be sufficiently separable in *m/z*-peaks feature space. Moreover, for more confident conclusions, a dataset for such task would require as many organs as possible for each of the species to be identified.

Even though there are thousands of higher plant species, at most only a few hundred are actively utilized in herbal medicine production. Such conditions readily allow liquid chromatography - low resolution mass-spectrometry in combination with machine learning to be successfully used for routine plant species identification task, given sufficient dataset. Unknown samples can be both in the forms of dry powder and alcohol-water extract or, likely, any form that retained relatively complete set of characteristic chemical compounds.

## Methods

### Sample preparation and LC-MS experiments

#### Chemicals and plant material

Methanol, ethanol, acetonitrile and formic acid were purchased from Merck (Germany). Deionized water was purified with Milli-Q water system (Millipore, Milford, MA, USA). Plant material was either collected by botanists or purchased from commercial suppliers.

#### Sample extraction

1 g of each plant was powdered in agate mortar and (a) sonicated for 1 hour in 10 mL of 70% EtOH, (b) sonicated for 1 hour in 10 mL of 70% MeOH, (c) sonicated for 3 hours in 10 mL of deionized water. 2 mL of crude extract were diluted 1:10 with 0.1% FA, centrifuged for 10 min (10000 g); 10 *μ*l of supernatant were subjected to LC-MS analysis.

#### LC-MS analysis

Parts of LC-MS experiment were conducted on 2 different platforms: (i) LCMS-IT-TOF (Shimadzu Corp, Japan) and (ii) Agilent QqQ 6430 (Agilent Technologies, USA), all equipped with ESI-source. Each platform was equipped with a binary solvent delivery unit, a degasser, an auto-sampler, and a column oven. MS data was collected in scan mode in range 100–900 *m*/*z* with both positive and negative polarities included. Default resolution settings were chosen for both platforms. Chromatography was performed on a Hypersil Gold aQ (Thermo scientific, USA) column (100 mm × 2.1 mmi.d, 1.9 *μ*m). Separation was performed with 0.1% aqueous formic acid (A) and 0.1% formic acid acetonitrile (B) according to the following gradient program: 0% to 95% B (0–12 min), 95% B (12–17 min), 95% to 0% B for 0.01 min and 0% B for 3 min. The temperature was set to 50 °C with flow rate 0.3 mL · min^−1^.

### Classification and feature extraction algorithms

#### Preprocessing

All LC-MS run files were converted into mzXML format. Then, chromatogram files were uploaded in Waters Progenesis QI software for peak picking procedure. Peak lists with and *t*_*R*_ tags for each sample were obtained and further converted according by the following procedure: *m*/*z* values of peaks were rounded to integers and only peaks with the highest abundance value for each *m*/*z* were chosen. This resulted in a vector with length 1600 (800 values for positive and negative polarity) for each respective sample.

Among 76 classes (explicit mapping between plant species and classes is given in Supplementary Fig. S1.6(a), 18 of them with fewer than 20 chromatograms were labeled as single class and used in training and validation sets as negative examples, which should not be designated by algorithm to any of the remaining 58 classes. Introducing such composed class, one can make classification algorithm to be more robust to inputs come from species unknown before.

#### Bayesian Networks

Feature space may be treated as random vector. In this case every sample may be considered as realization of such random vector. Thus it is possible to estimate joint probability distribution of its components. However, due to the curse of dimensionality this problem can not be resolved in a straightforward way. Different assumptions on random variables may ease this problem. The simplest one is statistical independence that leads to simple factorization of joint probabilistic density function (p.d.f.): $p({x}_{1},\,\ldots ,\,{x}_{m})=p({x}_{1})\cdot \ldots \cdot p({x}_{m})$. Here it was assumed that all *p*(*x*_1_) and joint p.d.f. exist. If one categorical variable *y* is added to this random vector $x=({x}_{1},\,\ldots ,\,{x}_{m})$, they become conditionally independent: $p({x}_{1},\,\ldots ,\,{x}_{m}|y)=p({x}_{1}|y)\cdot \ldots \cdot p({x}_{m}|y)$. Resulting probabilistic model known as naive Bayes^24^ is valid for using as classifier. But such assumption of conditional independence for all components of *x* is very restrictive, it is more natural to assume that there are interleaved dependencies across them. Any joint distribution $p({x}_{1},\,\ldots ,\,{x}_{m})$ may be decomposed into product (1) by repeatedly applying the product rule of probability^24^:

1 $$p({x}_{1},\,\ldots ,\,{x}_{m})=\prod _{i\mathrm{=1}}^{m}p({x}_{i}|pa[{x}_{i}]),$$

where *pa*[*x*_*i*_] is a designation for set of parents for *i*-th variable. Such decomposition is easy to visualize with graph where each vertex is associated with one variable (node), and edges represent relationships among them. Representations of joint distribution in form of graphs are known as probabilistic graphical models. Bayesian Networks are probabilistic graphical models with directed acyclic graph structure which represents internal conditional dependencies. If all random variables are drawn from discrete (or continuous) distribution, the corresponding Bayesian network is called discrete (continuous). If both discrete and continuous variables are used, such network is called hybrid Bayesian network. If one of variables is categorical and associated with class labels, then its value for each sample may be predicted with fitted Bayesian network. However, it is necessary to specify the structure of network first.

There exist different methods to learn graph structure from data^37^; most of them are computationally expensive. In case of very large dimensionality we used the Chow-Liu method^38^ to estimate a (sub-optimal) network. Input data were additionally preprocessed to keep 30% highest peaks and then transformed to binary masks of these highest peaks. All computations of models based on discrete Bayesian Networks were performed with the python pomegranate package^39^. Other approaches were used with input encoded by autoencoder which dramatically reduced number of variables. Scikit-learn^40^ implementation of naive Bayes classifier and implementation of hybrid Bayesian Networks in R package bnlearn^37^ were selected for use in this case. Interface for usage of R packages in python was provided by Rpy2 package. Visualization of network structure was done with NetworkX python package^41^.

#### Autoencoder

Autoencoder is a neural network which is designed to learn both direct transformation and the inverse of it. The output of autoencoder is to be as close to the input as possible. Such neural network may be utilized as adaptive feature extractor^30^. In this research, a feed-forward neural network with *N* = 2*n* layers was used where linear transformation of the latter *n* layers mirrored sizes of the former *n* ones. Nonlinearities were chosen to be consistent with non-negativity of LC-MS data (rectified linear unit, ReLU for the last layer and sigmoid function for others).

2 $$\hat{x}={f}_{N}({W}_{N}[\ldots \,{f}_{2}({W}_{2}[{f}_{1}({W}_{1}[x])])\ldots ]);\,N=2n,\,{f}_{i}(t)=\{\begin{array}{cc}\frac{1}{1+{e}^{-t}}, & {\rm{i}}{\rm{f}}\,i=\bar{1,\,N-1}\\ max\,(0,\,t), & {\rm{i}}{\rm{f}}\,i=N\end{array}$$

Parameters of the neural network were estimated via optimization of the specified objective function. Being more robust to outliers in comparison with minimal squared error loss, smoothed *l*_1_ loss function (also known as Huber loss) (3) was selected as a functional to be optimized by Adam method^42^. All computations were performed with the pyTorch package^43^.

3 $$l(x,\,\hat{x})=\sum _{i}\,{z}_{i},\,{z}_{i}=\{\begin{array}{cc}1/2({x}_{i}-{\hat{x}}_{i}{)}^{2}, & {\rm{i}}{\rm{f}}\,|{x}_{i}-{\hat{x}}_{i}| < 1\\ |{x}_{i}-{\hat{x}}_{i}|-1/2, & {\rm{o}}{\rm{t}}{\rm{h}}{\rm{e}}{\rm{r}}{\rm{w}}{\rm{i}}{\rm{s}}{\rm{e}}\end{array}$$

To prevent overfitting and to increase generalization ability of neural network, the number of output variables was made to decrease by a factor of 4 for each layer. Better performance was observed if the following pretraining procedure was used: at first, the simplest model with *N*_1_ = 2 is trained, then all learned layers are to be used in model with *N*_2_ = 4 as initialization, ending at desired level k with *N*_*k*_ = 2*k* = *N* layers. It was found that 3 encoding layers are sufficient to extract appropriate features to be used further in classifiers, namely, logistic regression, naive Bayes and general hybrid Bayesian network. It was additionally investigated how small the size of last encoding layer may be set without significant degrading of performance. Scikit-learn implementation^40^ of *l*_1_ regularized logistic regression was utilized in experiments. Other classifiers were adopted from packages specified in the above section.

#### Sparse non-negative Tucker decomposition

Original LC-MS data is a non-negative intensity function of 3 variables: mass-to-charge ratio (m/z), polarity and retention time. Initial preprocessing (peak picking) does not affect these coordinates but make the data sparse. In further preprocessing step retention time values were discarded, and data became function of 2 variables. Concatenation of multiple samples provides us additional axis. After quantization of m/z space, data is represented as 3-dimensional array (tensor), $T\in {{\mathbb{R}}}^{{N}_{{\rm{sample}}}\times {N}_{m/z}\times {N}_{{\rm{polarity}}}}$. On such data low-parametric tensor approximations are applicable to reveal its hidden structure. In Tucker decomposition (TD) data is parametrized by factor-matrices *A*, *B*, *C* and core tensor *G* of new shape:

4 $$\begin{array}{ccc}{T}_{ijk} & \approx  & \sum _{\alpha =1}^{{r}_{1}}\,\sum _{\beta =1}^{{r}_{2}}\,\sum _{\gamma =3}^{{r}_{3}}{g}_{\alpha \beta \gamma }{a}_{i\alpha }{b}_{j\beta }{c}_{k\gamma };\\ T & \approx  & |[G;\,A,\,B,\,C]|,\,G\in {{\mathbb{R}}}^{{r}_{1}\times {r}_{2}\times {r}_{3}},\,A\in {{\mathbb{R}}}^{{N}_{{\rm{s}}{\rm{a}}{\rm{m}}{\rm{p}}{\rm{l}}{\rm{e}}}\times {r}_{1}},\,B\in {{\mathbb{R}}}^{{N}_{m/z}\times {r}_{2}},\,C\in {{\mathbb{R}}}^{{N}_{{\rm{p}}{\rm{o}}{\rm{l}}{\rm{a}}{\rm{r}}{\rm{i}}{\rm{t}}{\rm{y}}}\times {r}_{3}}\end{array}$$

In (4) 3-dimensional decomposition is stated. Hyper parameters (*r*_1_, *r*_2_, *r*_3_) known as Tucker ranks affect sizes of factor matrices and core tensor. Tucker decomposition is not unique in general^44^, but it was proven that Tucker decomposition with sufficiently sparse non-negative parameters tends to be unique^45^. Our dataset adheres to these constraints, thus the algorithm for computing sparse non-negative Tucker decomposition (SNTD) was implemented as described in^46^.

Estimated factor matrices were used for classification. Because any new sample is a matrix of *N*_m/z_ × *N*_polarity_ size, factor-matrix *A* for sample axis was rejected from estimation procedure, which is equivalent to enforcing it to be identity matrix *I*. The resulting optimization task to be solved to estimate parameters of decomposition is

5 $$\begin{array}{cc}\mathop{min}\limits_{G,B,C} & {\parallel |[G;\,I,\,B,\,C]|-T\parallel }_{F}^{2}+{\lambda }_{G}{\parallel {\rm{v}}{\rm{e}}{\rm{c}}(G)\parallel }_{1}+{\lambda }_{B}{\parallel {\rm{v}}{\rm{e}}{\rm{c}}(B)\parallel }_{1}+{\lambda }_{C}{\parallel {\rm{v}}{\rm{e}}{\rm{c}}(C)\parallel }_{1}\\ {\rm{s}}.{\rm{t}}. & G\in {{\mathbb{R}}}_{\ge 0}^{{N}_{{\rm{s}}{\rm{a}}{\rm{m}}{\rm{p}}{\rm{l}}{\rm{e}}}\times {r}_{2}\times {r}_{3}},\,B\in {{\mathbb{R}}}_{\ge 0}^{{N}_{m/z}\times {r}_{2}},\,C\in {{\mathbb{R}}}_{\ge 0}^{{N}_{{\rm{p}}{\rm{o}}{\rm{l}}{\rm{a}}{\rm{r}}{\rm{i}}{\rm{t}}{\rm{y}}}\times {r}_{3}},\end{array}$$

where *λ*_*G*_, *λ*_*B*_, *λ*_*C*_ are penalties for insufficient sparseness, ${\rm{vec}}(\,\cdot \,)$ is a vectorization of input, ${{\mathbb{R}}}_{\ge 0}$ - non-negative real space.

For Tucker decomposition the rank of polarity axis was set to 2, and the rank of m/z space was selected to be the same for all classes. Rank selection of the latter space was performed via grid search.

Classification procedure was organized as follows: (1) compute factor matrices of m/z and polarity axes for samples of each identity; (2) multiply every input to be classified by inverse of polarity factor-matrix; (3) compute vector of distances from column space of processed input to column spaces of m/z factor-matrices of each identity; (4) select identity with minimal distance as predicted label. To compute distances, two metrics were selected: principal angle^47^ (similar approach to one used in^48^) and distance correlation^49^, the latter was computed with python dcor package.

#### Sparse non-negative matrix factorization

In matrix factorization (MF) problem it is required to find such two matrices *S* and *M* the product of which approximates original data matrix *X* as accurately as possible, $X\approx S{M}^{T}$. This task is related to estimation of basis in a linear space. Columns of matrix *M* provide essential description of samples from a given class. As in sparse NTD, we measure distances between linear spans of components extracted by NMF algorithm and an input sample instead of direct projection of inputs. Class with minimal such a distance would be assigned as result for a query. One of the basic assumptions is that such decomposition contains low number of parameters, i.e. what is usually referred to as low-rank decompositions. Another assumption concerns the properties of parameters. Like in the SNTD approach, one may assume that matrices *S* and *M* are sparse and have non-negative elements, leading to sparse non-negative matrix factorization (SNMF).

It is worth noting that the NTD approach considered above may be viewed as a special case of NMF with separated polarity and m/z modes:

6 $${T}_{\mathrm{(1)}}\approx \mathop{\underbrace{{G}_{\mathrm{(1)}}}}\limits_{S}\mathop{\underbrace{{(C\otimes B)}^{T}}}\limits_{{M}^{T}},\,{T}_{\mathrm{(1)}}\in {{\mathbb{R}}}^{{N}_{{\rm{sample}}}\times {N}_{m/z}\cdot {N}_{{\rm{polarity}}}},\,{G}_{\mathrm{(1)}}\in {{\mathbb{R}}}^{{N}_{{\rm{sample}}}\times \hat{r}},\,\hat{r}={r}_{2}\cdot {r}_{3}$$

where $\otimes$ denotes a Kronecker product between matrices, and *T*_(1)_, *G*_(1)_ are sample-mode unfoldings (matrizations) of the tensors *T* and *G*. As in Tucker decomposition, the rank of merged axes was selected by inspecting accuracy changes in 5-fold cross validation scheme (see Fig. 2).

### Cross validation and performance scoring

All samples from the dataset were partitioned into 5 splits of train and test parts containing 80% and 20% of the data (5-fold cross validation). For the sake of uniformity, identical splitting on train and test parts was used for all algorithms with maintaining constant fraction of samples per each class in both parts at every fold. All results presented in Table 1 were computed with 5-times repeated 5-fold cross validation.

To measure performance of the algorithms standard metrics were chosen: accuracy and F-measure. The former indicates a fraction of correctly predicted labels. The latter is defined for binary classification with “negative” and “positive” samples as harmonic average of precision (ability to avoid predicting negative sample as positive) and recall (ability to correctly classify all positive samples). In multiclass task this index may be measured independently for each class where “positive” label means that sample drawn from current class, “negative” - from any other. Taking into account the unbalanced quantities of samples per class, weighted average was used.

Additionally, computational times are provided to give a reference point for computational costs for each approach. Packages^50–55^ were used as well as the ones mentioned in the above sections.

## Electronic supplementary material

Supplementary Figure S1

## Data Availability

All implemented algorithms and processed data are available via GitHub repository, https://github.com/kharyuk/chemfin-plasp/, and Docker repository, https://hub.docker.com/r/kharyuk/chemfin-plasp/. Computational experiments are organized in Jupyter Notebooks which are numbered according to suggested order of launching. Data is transited between them through generation of files with intermediary results. Most of the raw data (all 2263 files from original dataset and 18 out of 44 files from Test2) can be found in two Mendeley repositories, part 1 10.17632/bsmy8yj52s.3, part 2 10.17632/fnh2gy4nfy.1 and MetaboLights repository, https://www.ebi.ac.uk/metabolights/mtbls688.
