# Author Correction: Employing fingerprinting of medicinal plants by means of LC-MS and machine learning for species identification task

**DOI:** 10.1038/s41598-020-67201-4

**Published:** 2020-07-08

**Authors:** Pavel Kharyuk, Dmitry Nazarenko, Ivan Oseledets, Igor Rodin, Oleg Shpigun, Andrey Tsitsilin, Mikhail Lavrentyev

**Affiliations:** 1 Skolkovo Institute of Science and Technology, Center for Computational and Data-Intensive Science and Engineering, Moscow, 143026 Russia; 2 Institute of Numerical Mathematics of the Russian Academy of Sciences, Moscow, 119991 Russia; 3 Lomonosov Moscow State University, Faculty of Chemistry, Moscow, 119991 Russia; 4 All-Russian Research Institute of Medicinal and Aromatic Plants (VILAR), Moscow, 117216 Russia; 5 Saratov State University, Department of Botanics and Ecology, Saratov, 410012 Russia

Correction to: *Scientific Reports* 10.1038/s41598-018-35399-z, published online 19 November 2018

This Article contained errors.

Following the publication of this Article, the authors discovered unintentional train-test leakage in the machine learning experiment. This was caused by the authors not taking into account highly correlated LC-MS repletions of individual physical samples. This is now corrected.

In the abstract:

“Even with elimination of all retention time values accuracies of up to 96% and 92% were achieved on validation set for plant species and plant organ identification respectively.”

now reads:

“Even with elimination of all retention time values accuracies of up to around 85% were achieved on validation set for plant species and plant organ identification.”

In the Results:

“Encoded data vectors with 25 variables were used to train logistic regression and continuous Bayes classifiers (bothNaive Bayes and hybrid BayesianNetwork) with resulting identification accuracy of 96% and 84–87% on Test 1 respectively. All abovementioned models showed accuracy of 68–77% on Test 2.”

now reads:

“Encoded data vectors with 25 variables were used to train logistic regression and continuous Bayes classifiers (both Naive Bayes and hybrid Bayesian Network) with resulting identification accuracy of 85% and 68-69% on Test 1 respectively. All of the above mentioned models showed accuracy of 68-75% on Test 2.”

“According to the Table 1 Part 1, classifier based on Tucker decomposition with principal angle distance measure performs well (93% and 86% respectively for Test 1 and Test 2).”

now reads:

“According to the Table Table 1 Part 1 Part 1, classifier based on Tucker decomposition with principal angle distance measure performs well (78% and 84% respectively for Test 1 and Test 2).”

In the Discussion:

“The most obvious increase was shown by BN on Test 2, where emergence of correct labels in Top5 jumped by more than 20% compared to “winner takes all” approach. Although exact accuracy values may drop when using larger and more diverse datasets, this shows great potential of discrete BNs in such applications. All in all, TopN representation can be considered a more preferable way of output – narrowing possible candidates to 3–5 with 95% or more accuracy can be more beneficial than 80% accurate single candidate species.”

now reads:

“The most obvious increase was shown by bayesian networks on Test 2, where emergence of correct labels in Top5 jumped by around 20% compared to “winner takes all” approach. Although exact accuracy values may drop when using larger and more diverse datasets, this shows great potential of BNs in such applications. All in all, TopN representation can be considered a more preferable way of output – narrowing possible candidates to 3-5 with 90% accuracy can be more beneficial than 75% accurate single candidate species.”

“Algorithms showed high distinguishing ability between most classes (up to 92% accuracy), excluding very similar pair of classes (roots, roots and rhizomes).”

now reads:

“Algorithms showed high distinguishing ability between most classes (up to 86% accuracy), excluding very similar pair of classes (roots, roots and rhizomes).”

Additionally, as a result of these errors, Figures 2, 6, Table 1 and the Supplementary Figure file S1 have been corrected in the original HTML and PDF of this Article. The original versions of Figures 2, 6 and Table 1 are reproduced below as Figure 1, Figure 2 and Table 1 respectively. The original version of Supplementary Figure 1 is included as a Supplementary File in this notice.

**Figure 1:**
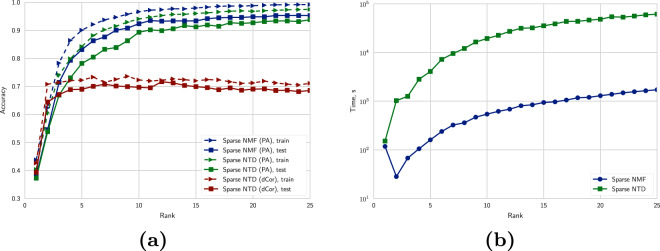
.

**Figure 2:**
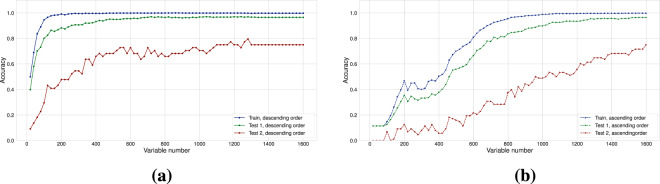
.

**Table 1:** Comparative characteristics of implemented approaches.

Part 1. Results for “winner takes all” strategy. Prediction times are written per one sample. For classifiers based on features spaces learned with autoencoder additional times for estimation of autoencoder parameters are given in parentheses										
Method	Accuracy, %			F1, %			Time			
	Train	Test 1	Test 2	Train	Test 1	Test 2	Training	Prediction		
Logistic regression (autoencoded)	99.7	96.5	72.7	99.7	96.4	77.3	1 m 16 s (+1 h 30 m)	0.06 ms		
Naive Bayes (autoencoded)	89.6	84.5	77.3	89.8	84.6	83.3	8 ms (+1 h 30 m)	0.02 ms		
Hybrid BN (autoencoded)	92.2	87.2	68.2	92.4	87.1	74.8	50 m 47 s (+1 h 30 m)	1.8 ms		
Large discrete BN	—	90.0	72.7	—	90.0	81.0	3 m 14 s	9 m		
Sparse NTD (principal angle)	97.6	93.4	86.4	97.6	93.3	91.1	18 h 19 m	1.1 s		
Sparse NMF (principal angle)	99.2	94.8	81.8	99.2	94.9	84.1	28 m 46 s	1.1 s		
**Part 2. TopN approach. Output is considered to be accurate when correct label is present in TopN results**.										
**Method**	**Accuracy, %**									
	**Test 1**					**Test 2**				
	**Top1**	**Top2**	**Top3**	**Top4**	**Top5**	**Top1**	**Top2**	**Top3**	**Top4**	**Top5**
Logistic regression (autoencoded)	96.5	98.5	99.1	99.3	99.5	72.7	79.6	84.1	84.1	86.4
Naive Bayes (autoencoded)	84.5	91.6	94.2	95.7	96.7	77.3	86.4	88.6	93.2	93.2
Large discrete BN	90.0	93.8	95.1	95.1	95.3	72.7	81.8	88.6	90.9	93.2
Sparse NTD (principal angle)	93.4	95.9	96.6	97.1	97.4	86.4	88.6	90.9	90.9	93.2
Sparse NMF (principal angle)	94.8	96.2	96.5	96.9	97.1	81.8	84.1	86.4	86.4	88.6
**Part 3. Plant organ identification**.										
**Method**	**Accuracy, %**			**F1, %**						
	**Train**	**Test 1**	**Test 2**	**Train**	**Test 1**	**Test 2**				
Logistic regression (autoencoded)	86.3	83.1	68.2	86.1	82.6	64.1				
Naive Bayes (autoencoded)	76.6	74.7	63.6	76.1	74.2	58.3				
Large discrete BN	76.4	74.7	65.9	76.1	73.9	63.0				
Sparse NTD (principal angle)	89.9	87.6	86.4	90.3	87.9	87.7				
Sparse NMF (principal angle)	96.2	94.2	84.1	96.3	94.3	84.6				

Test 2 is independent from Train/Test 1 parts. In Part 1 and Part 3 all values presented are medians across 5-times repeated 5-fold cross validation runs. In Part 2 the same partitioning was used but final results were computed as top-N’s (see Supplementary S1.2).

These errors have now been corrected in the PDF and HTML versions of the Article, and in the accompanying Supplementary Information file.

## Supplementary information

Supplementary Information.

