# Supplementary material for: Employing fingerprinting of medicinal plants by means of LC-MS and machine learning for species identification task
Source: Sci Rep. 2018 Nov 19;8:17053. doi: 10.1038/s41598-018-35399-z (PMC6243014; doi:10.1038/s41598-018-35399-z)
Supplement: Supplementary file 1 — Supplementary Figure S1 [file 41598_2018_35399_MOESM1_ESM.pdf]

Employing fingerprinting of medicinal plants by means of LC-MS and machine learning for species identification task

Pavel Kharyuk, Dmitry Nazarenko, Ivan Oseledets, Igor Rodin, Oleg Shpigun, Andrey Tsitsilin, Mikhail Lavrentyev

Contents

1 Confusion matrices **1**

2 Top5 predictions **14**

3 Hierarchical clustering analysis (HCA) **20**

4 Sparse non-negative components **21**

5 Autoencoder: structure, t-SNE plots and selection of last layer size **24**

6 Dataset **27**

7 Confusion matrices for prediction of plant parts **29**

8 Github repository structure. **32**

1 Confusion matrices

In this section we provide confusion matrices measured on test1 part as mean and median of 5 times repeated 5-fold cross validation (25 runs in total). Columns: predicted labels; rows: true labels.

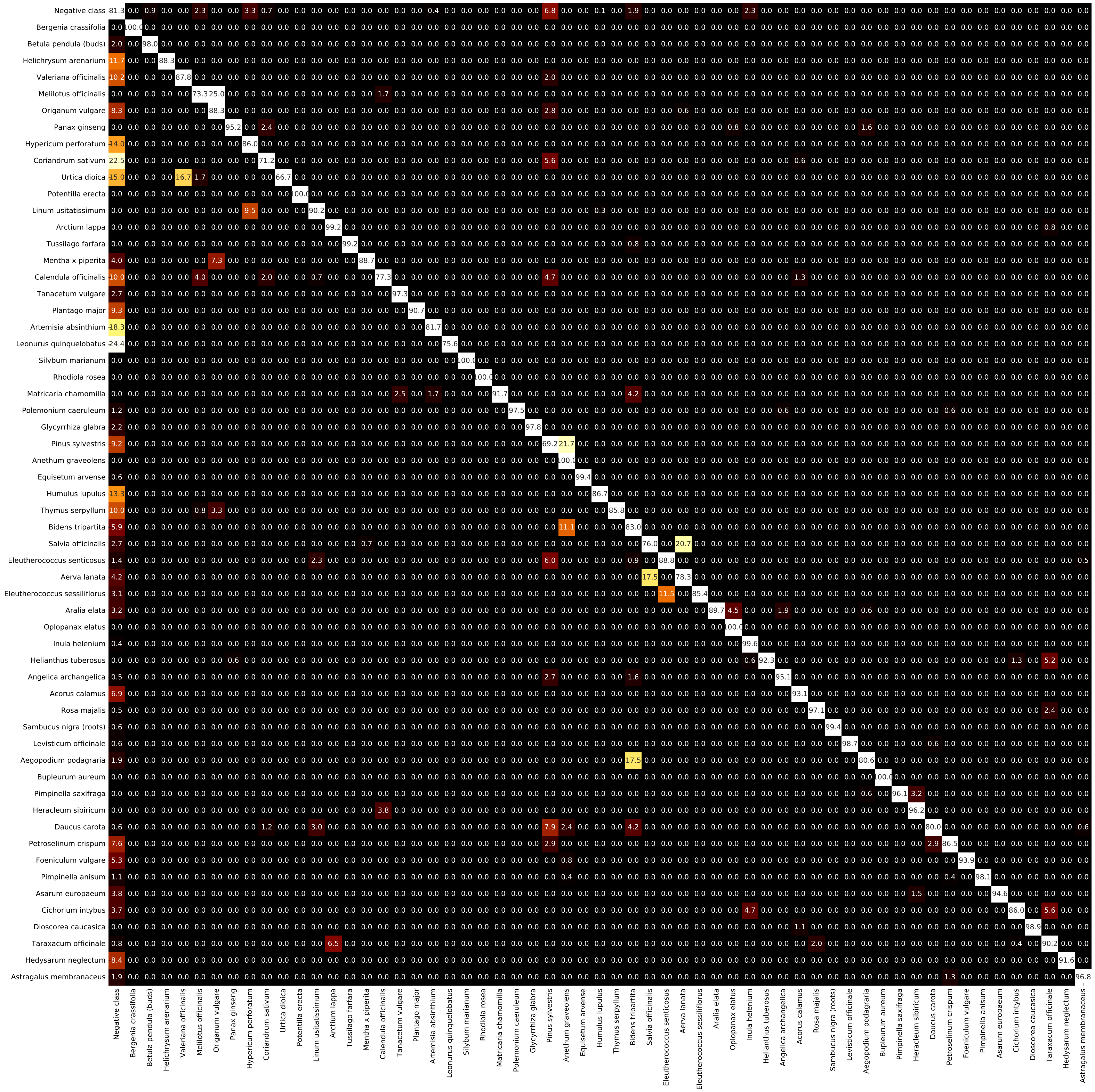

Figure S1.1(a): Mean confusion matrix for Large discrete Bayesian Network

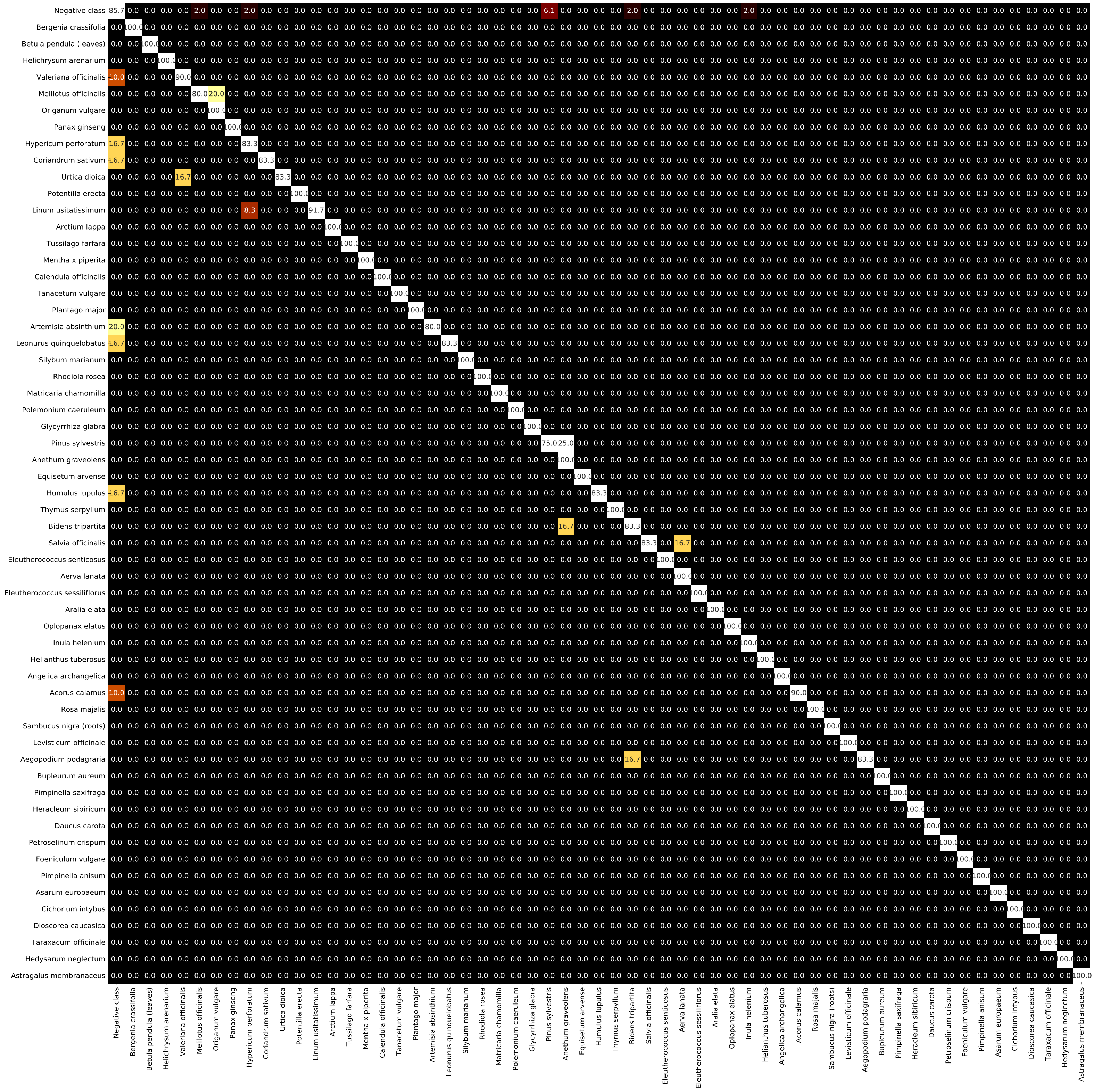

Figure S1.1(b): Median confusion matrix for Large discrete Bayesian Network

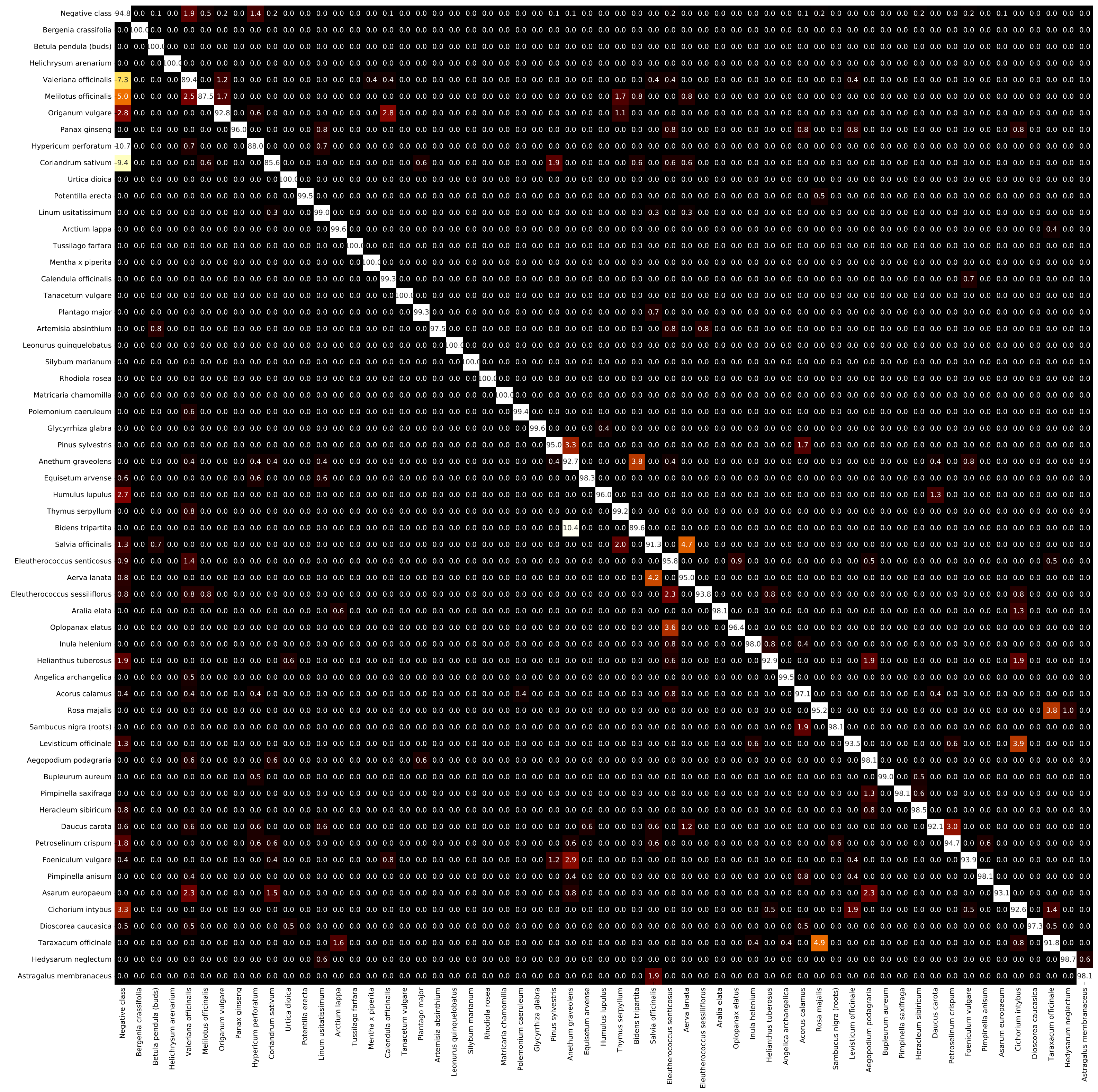

Figure S1.1(c): Mean confusion matrix for Logistic Regression trained on autoencoded feature space

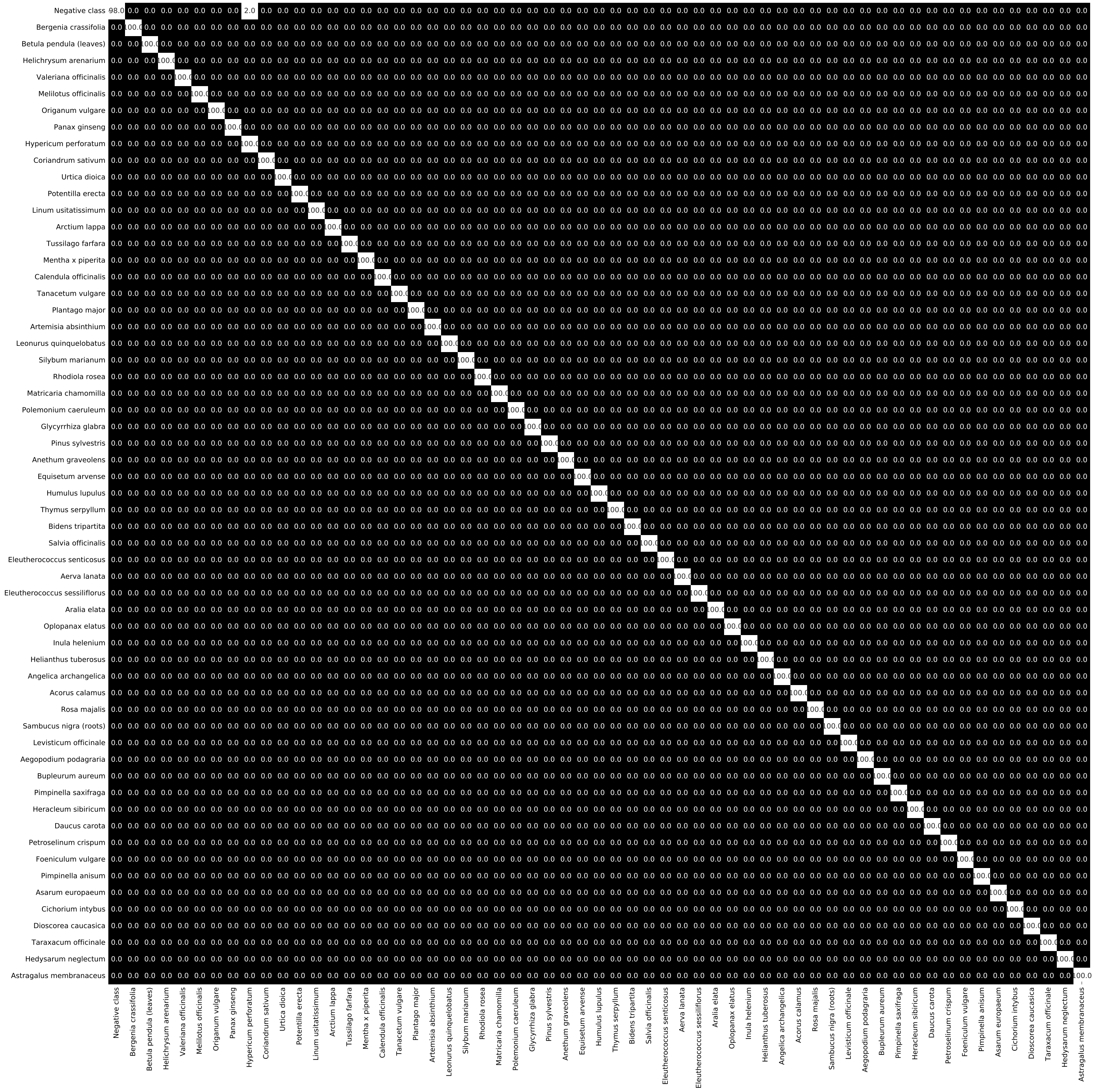

Figure S1.1(d): Median confusion matrix for Logistic Regression trained on autoencoded feature space

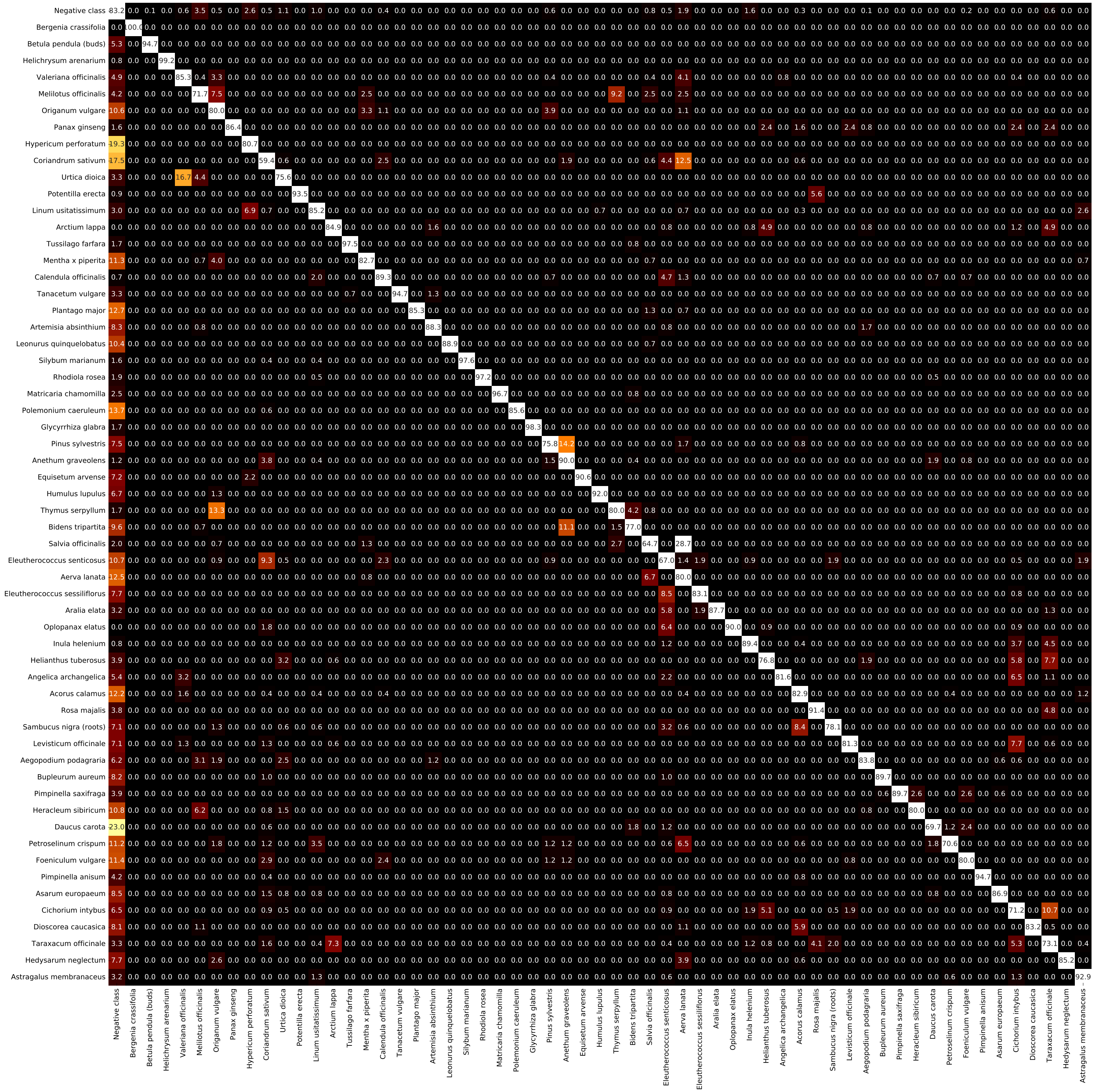

Figure S1.1(e): Mean confusion matrix for Naive Bayes classifier trained on autoencoded feature space

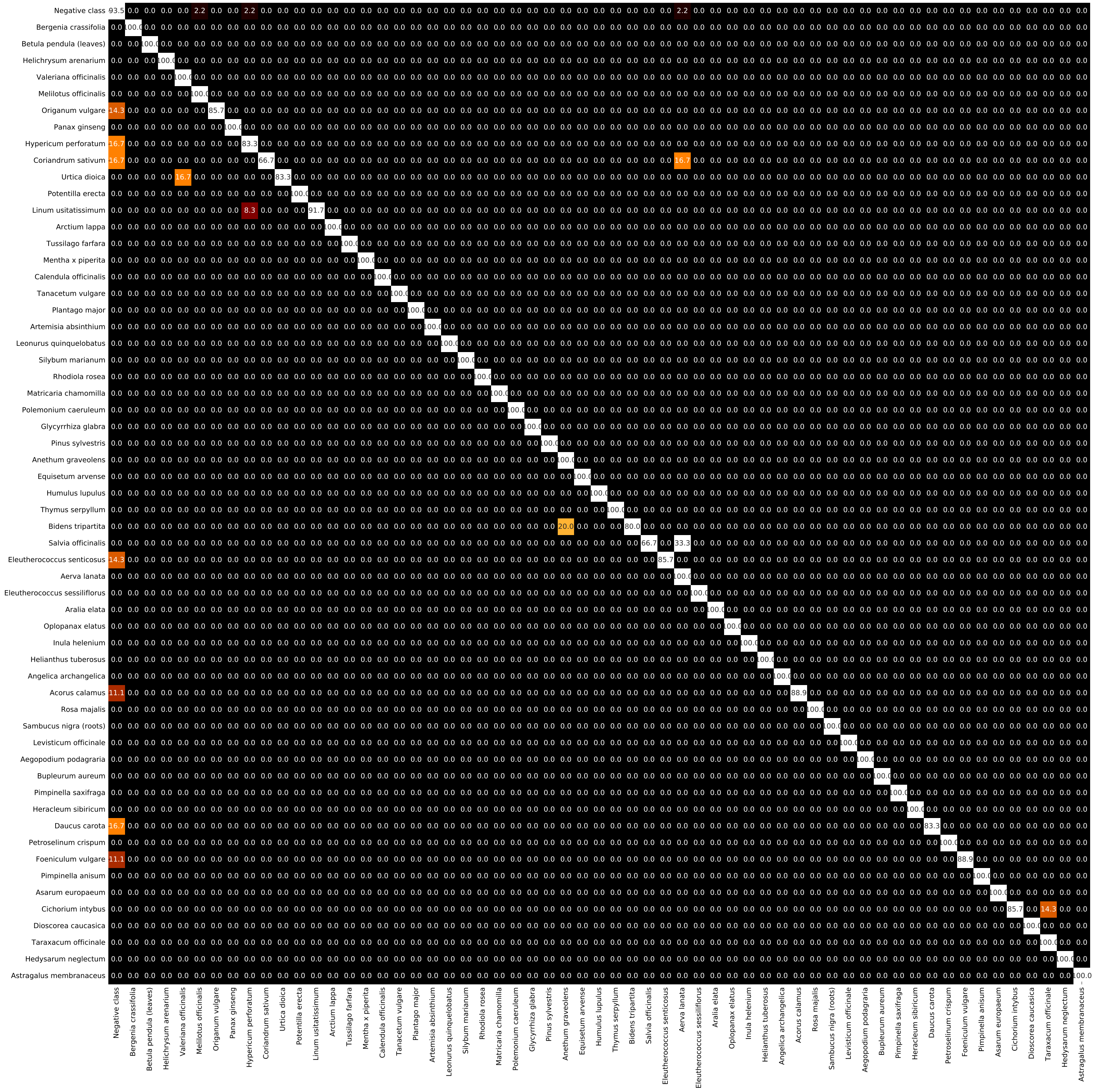

Figure S1.1(f): Median confusion matrix for Naive Bayes classifier trained on autoencoded feature space

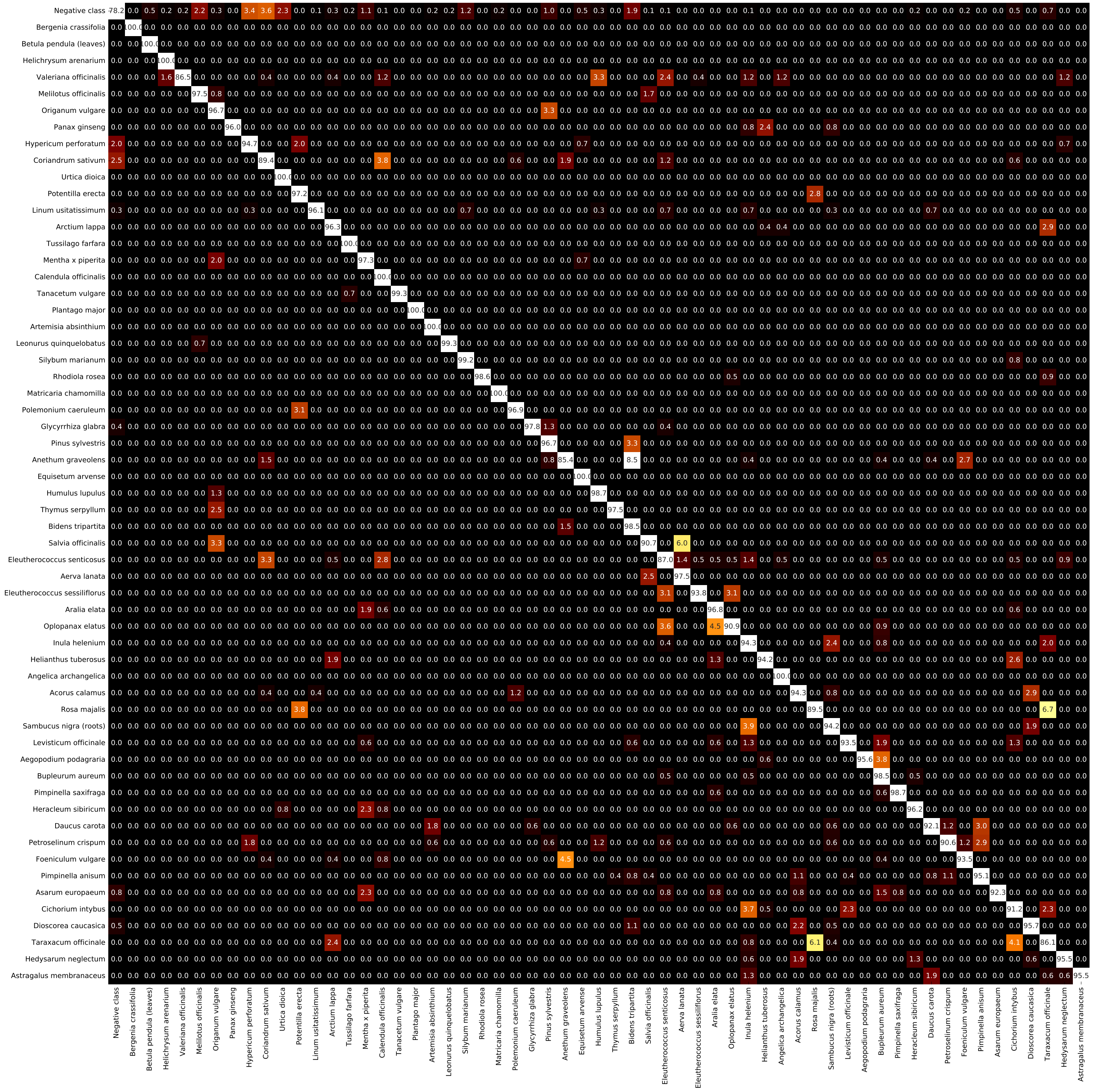

Figure S1.1(g): Mean confusion matrix for sparse non-negative Tucker decomposition based classifier (with principal angle)

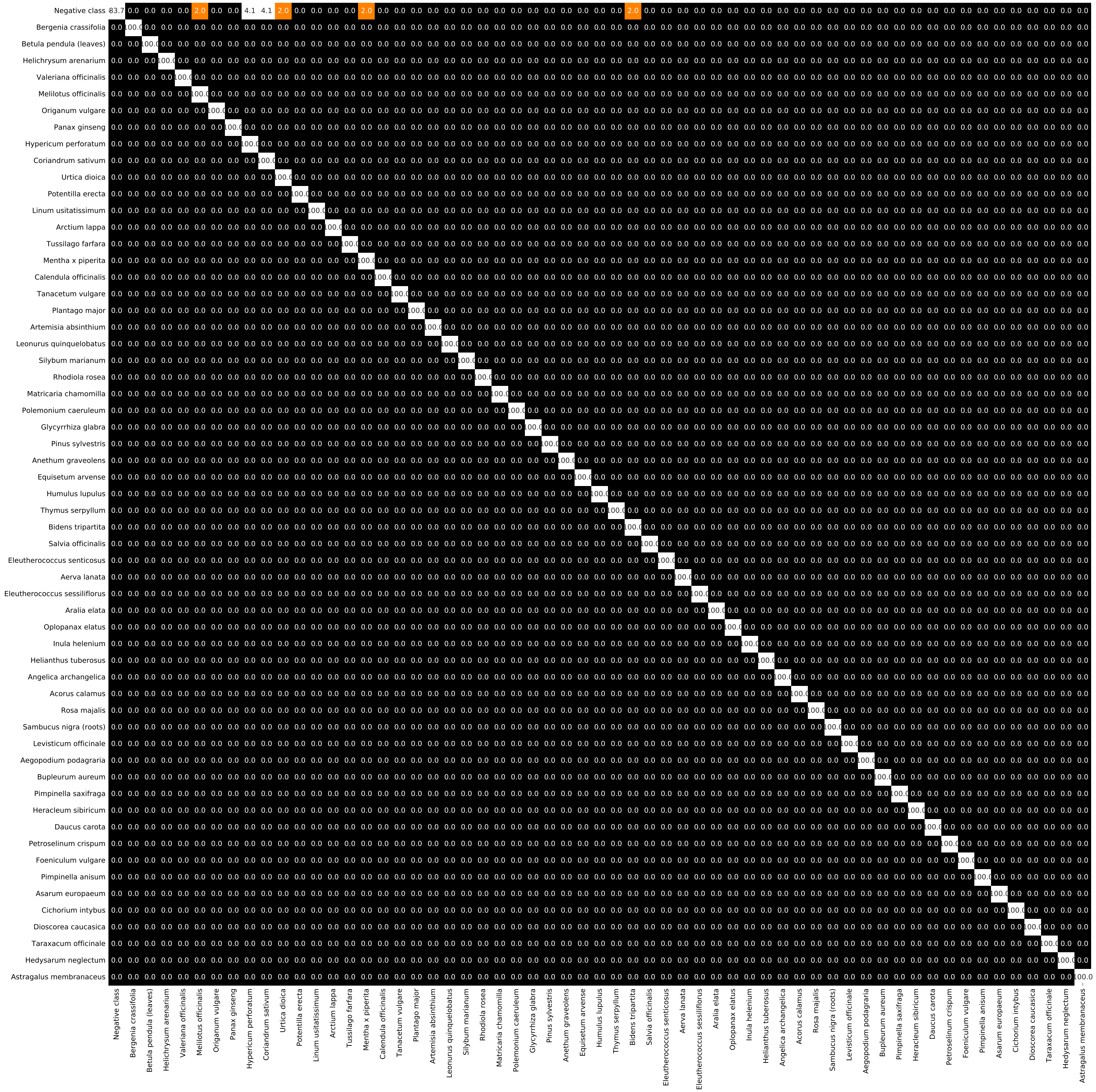

Figure S1.1(h): Median confusion matrix for sparse non-negative Tucker decomposition based classifier (with principal angle)

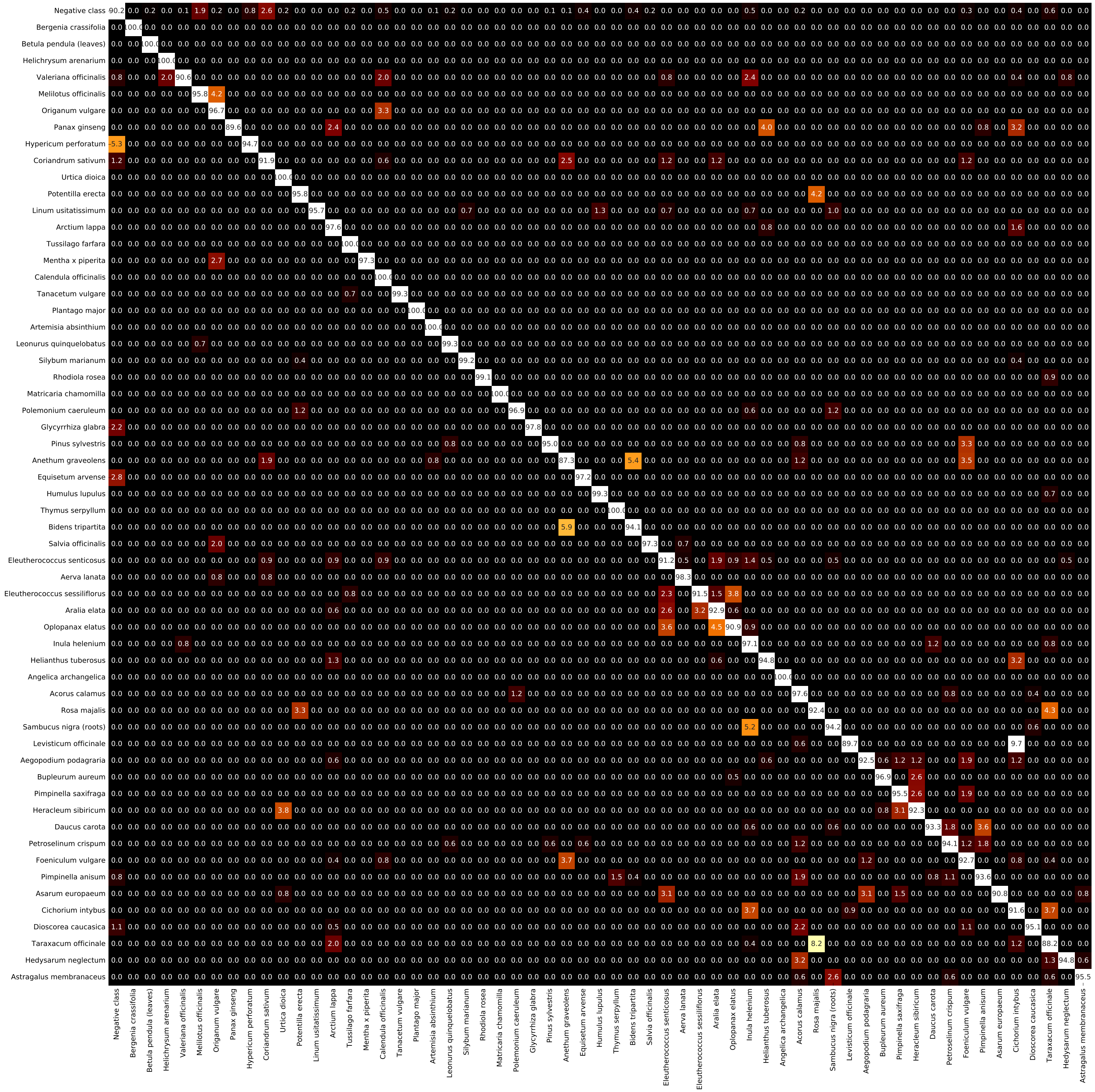

Figure S1.1(i): Mean confusion matrix for sparse non-negative matrix factorization based classifier (with principal angle)

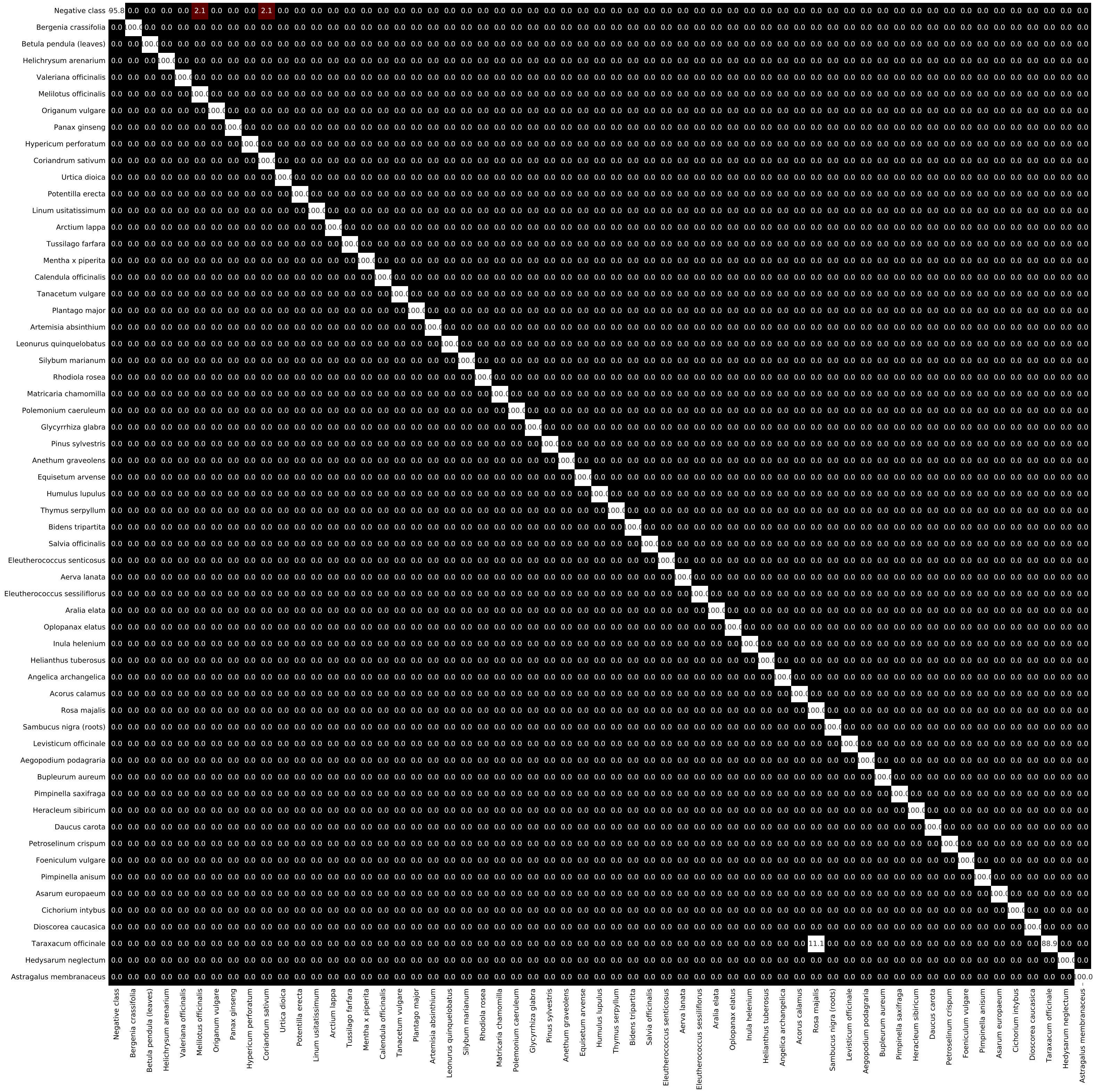

Figure S1.1(j): Median confusion matrix for sparse non-negative matrix factorization based classifier (with principal angle)

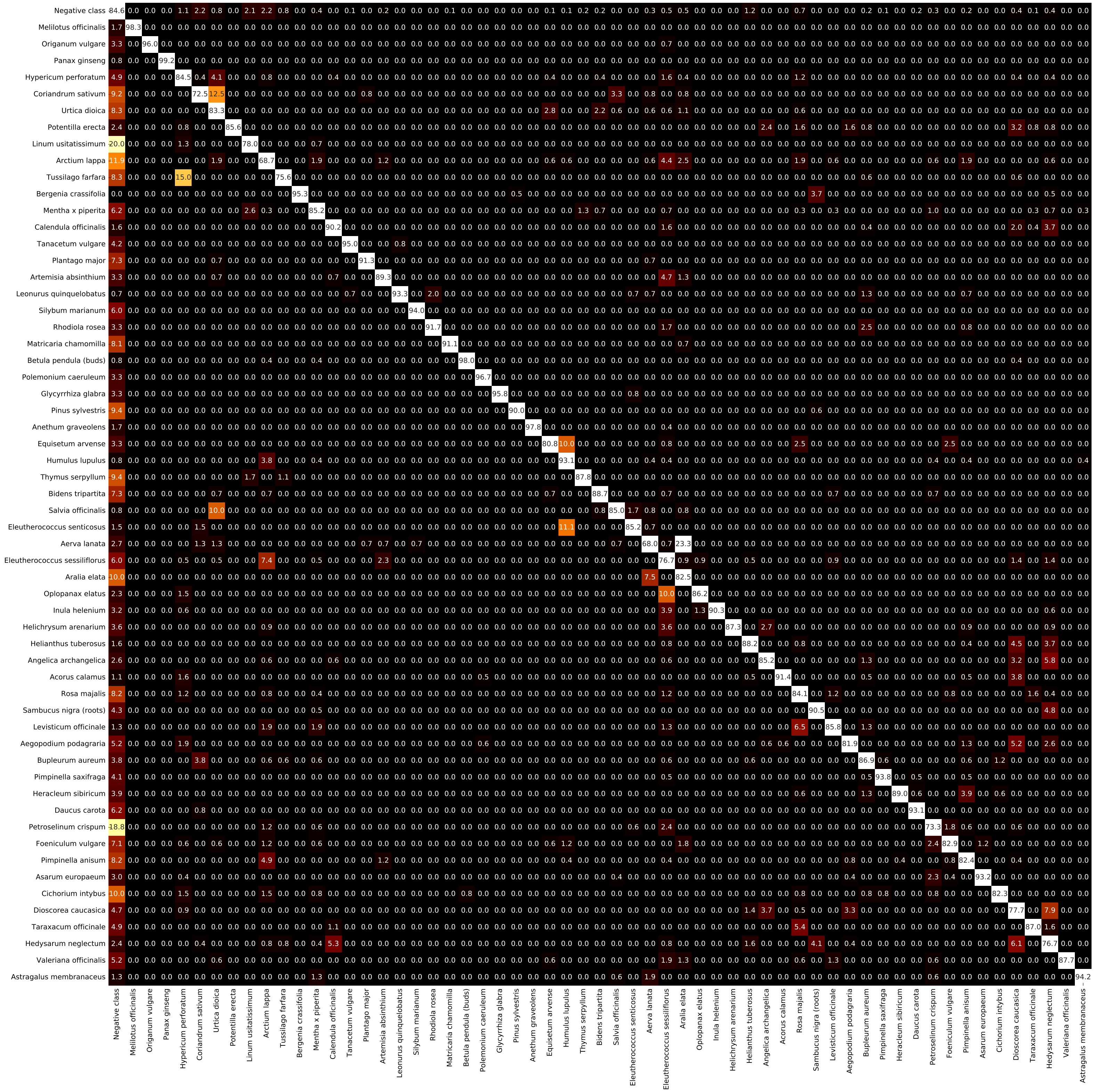

Figure S1.1(k): Mean confusion matrix for Hybrid Bayesian Network-based classifier trained on autoencoded feature space

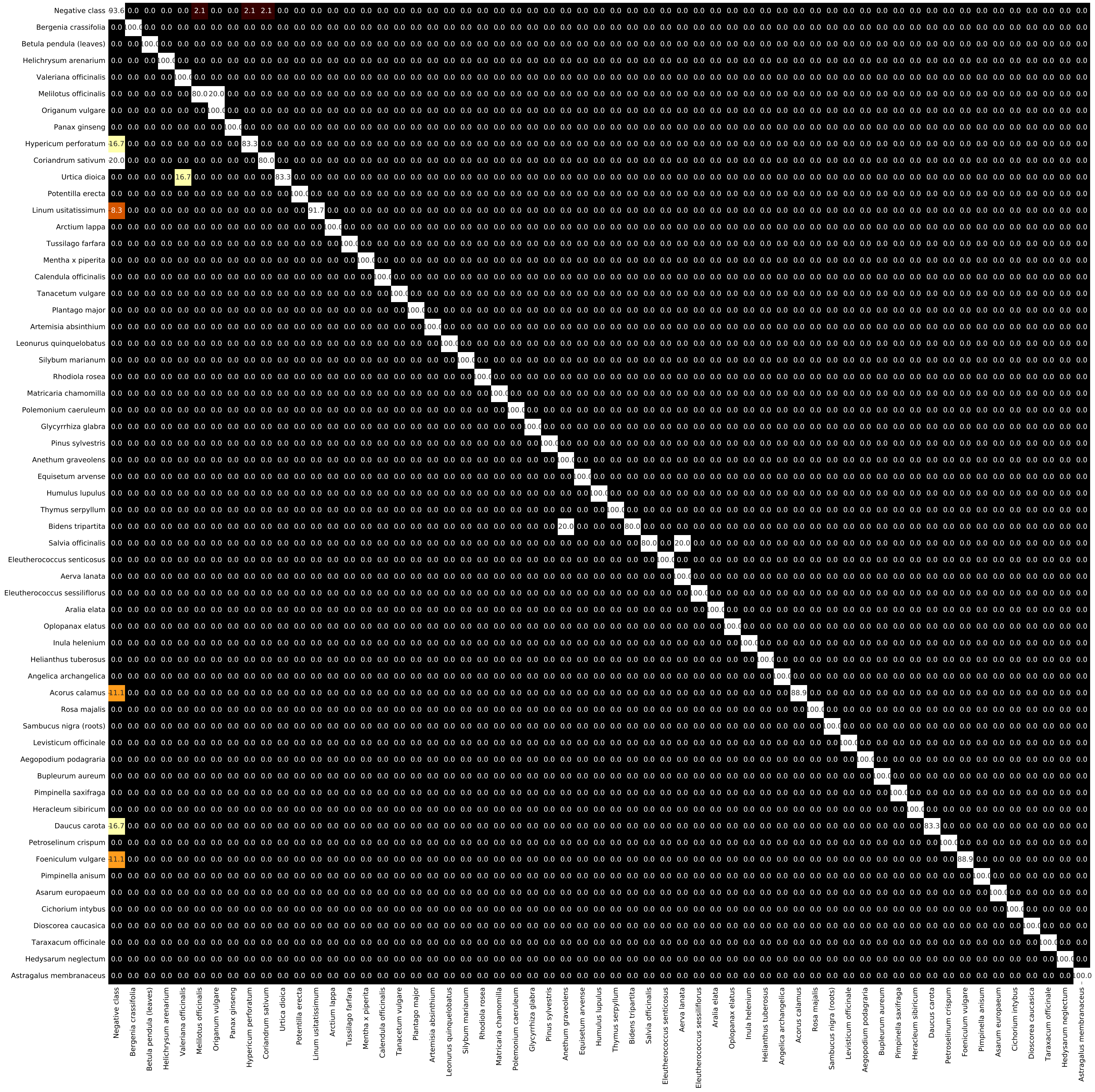

Figure S1.1(l): Median confusion matrix for Hybrid Bayesian Network-based classifier trained on autoencoded feature space

2 Top5 predictions

Top5 prediction results for each sample of a class (inside each fold) were extracted and pooled together. Top5 frequent results from this list were selected and this top5s were then pooled from all 25 parts of CV (5 repetitions of 5 folds). Resulting top5 list was selected as “neighbors” for that class. Columns: true labels; rows: “neighbors”.

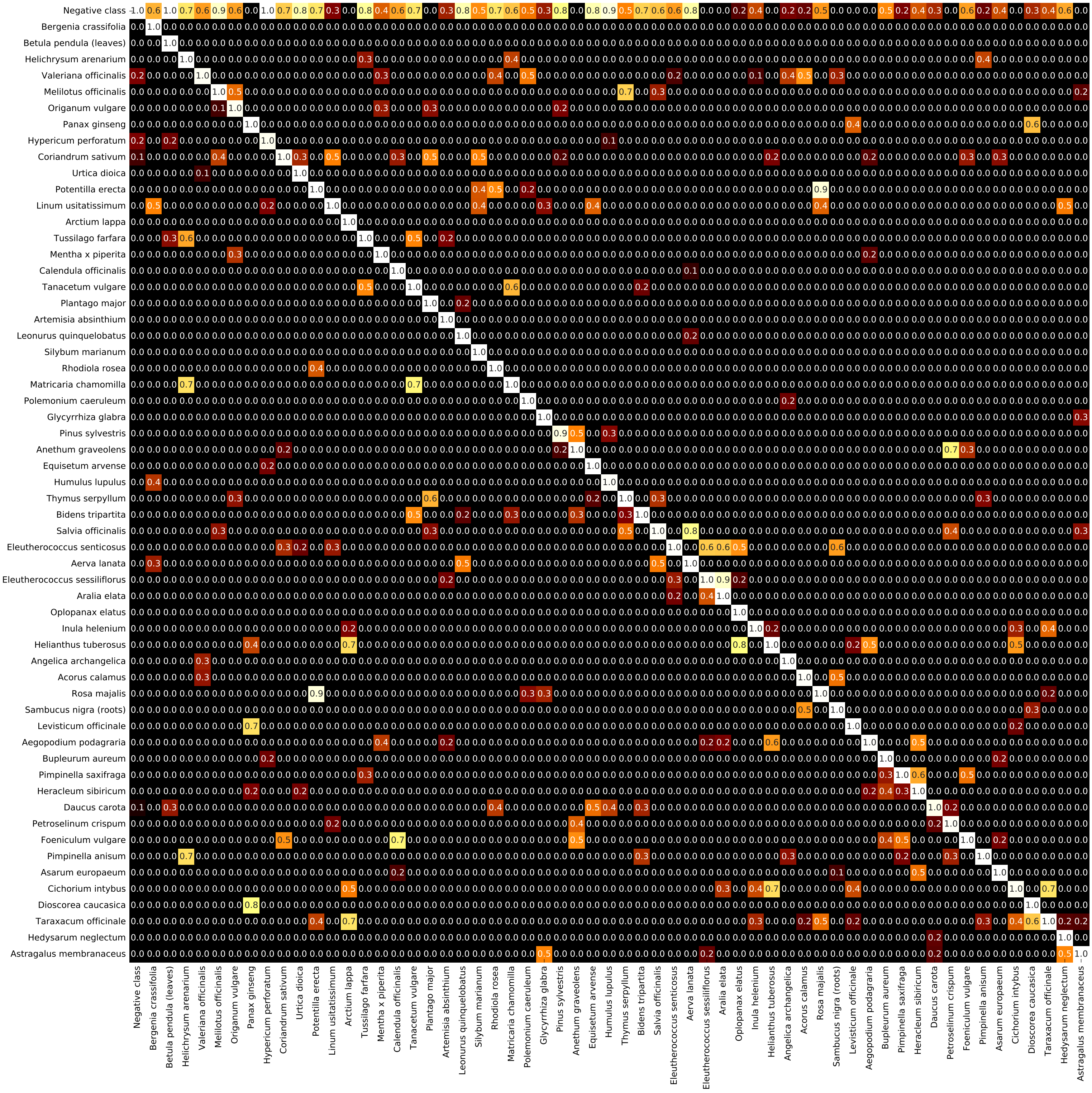

Figure S1.2(a): Logistic regression on features encoded by autoencoder

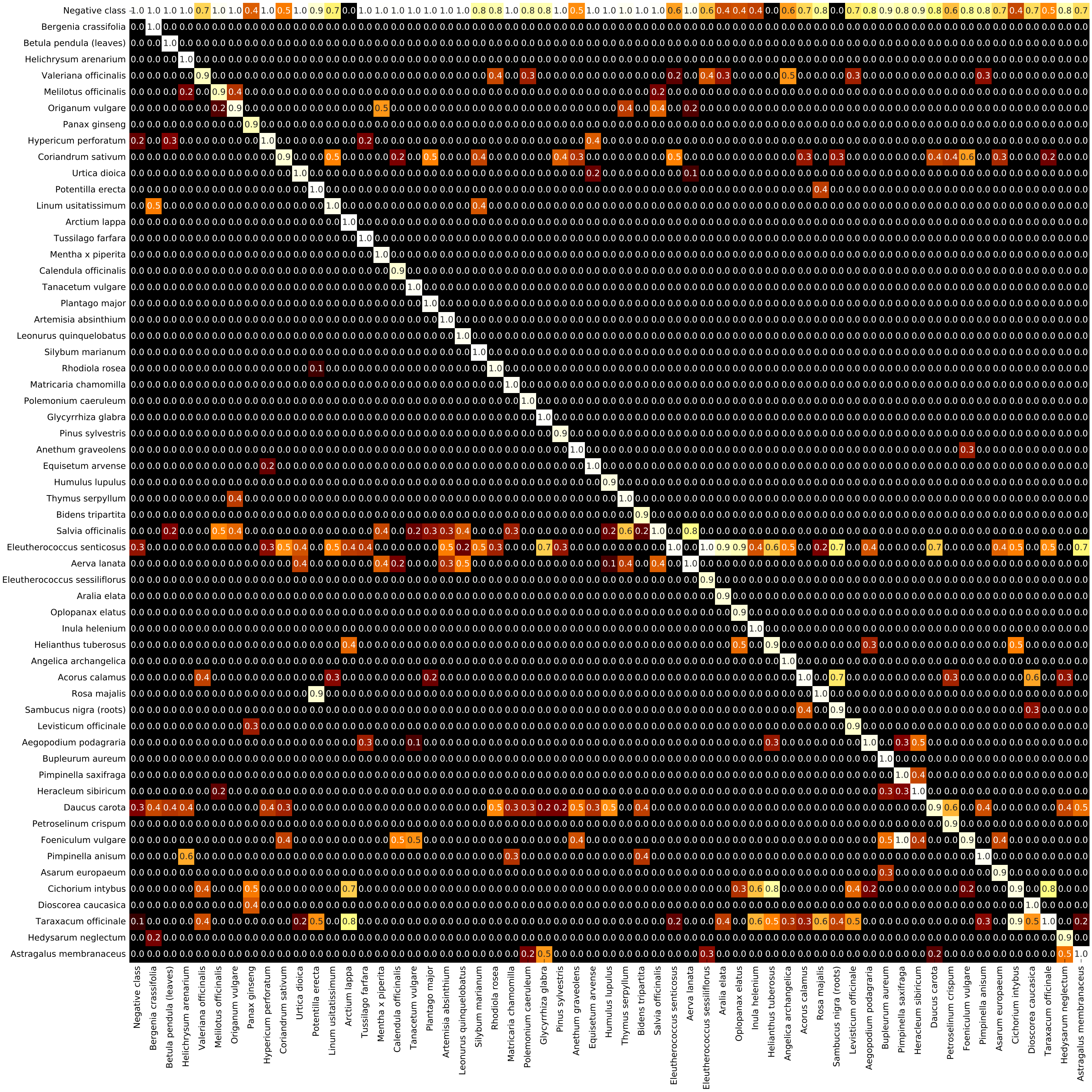

Figure S1.2(b): Naive Bayes classifier on features encoded by autoencoder

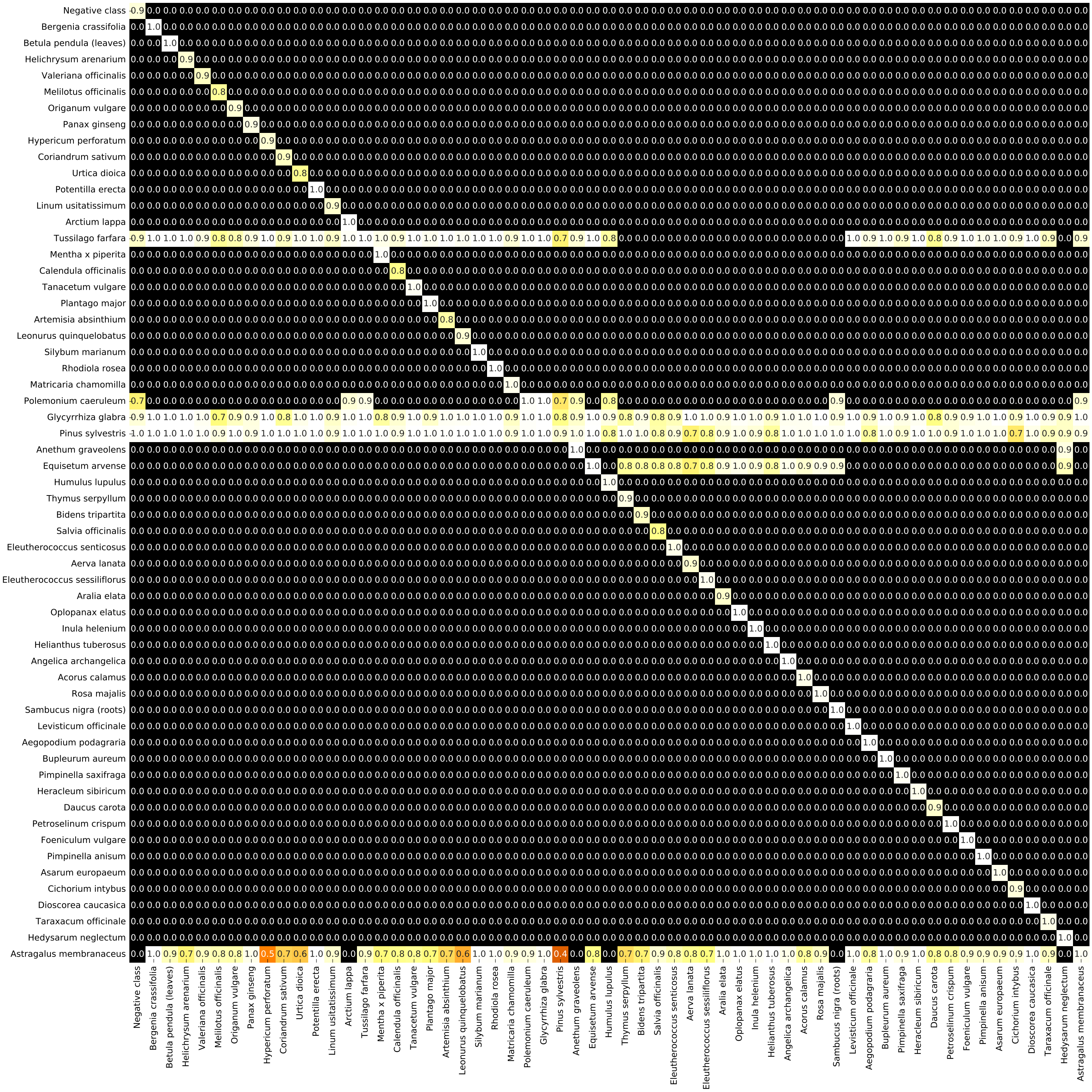

Figure S1.2(c): Large discrete Bayesian Network on binarized features

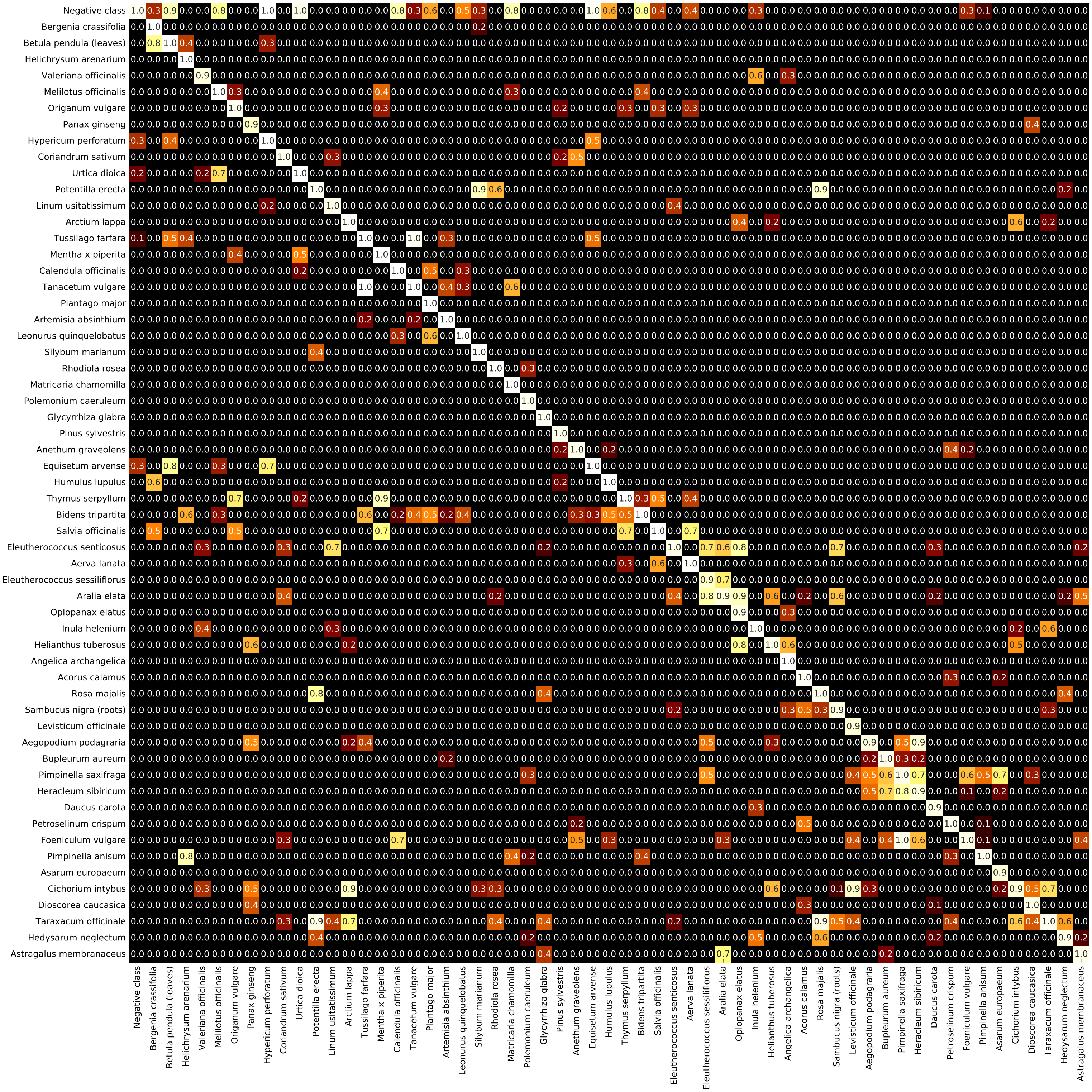

Figure S1.2(d): Sparse NMF classifier

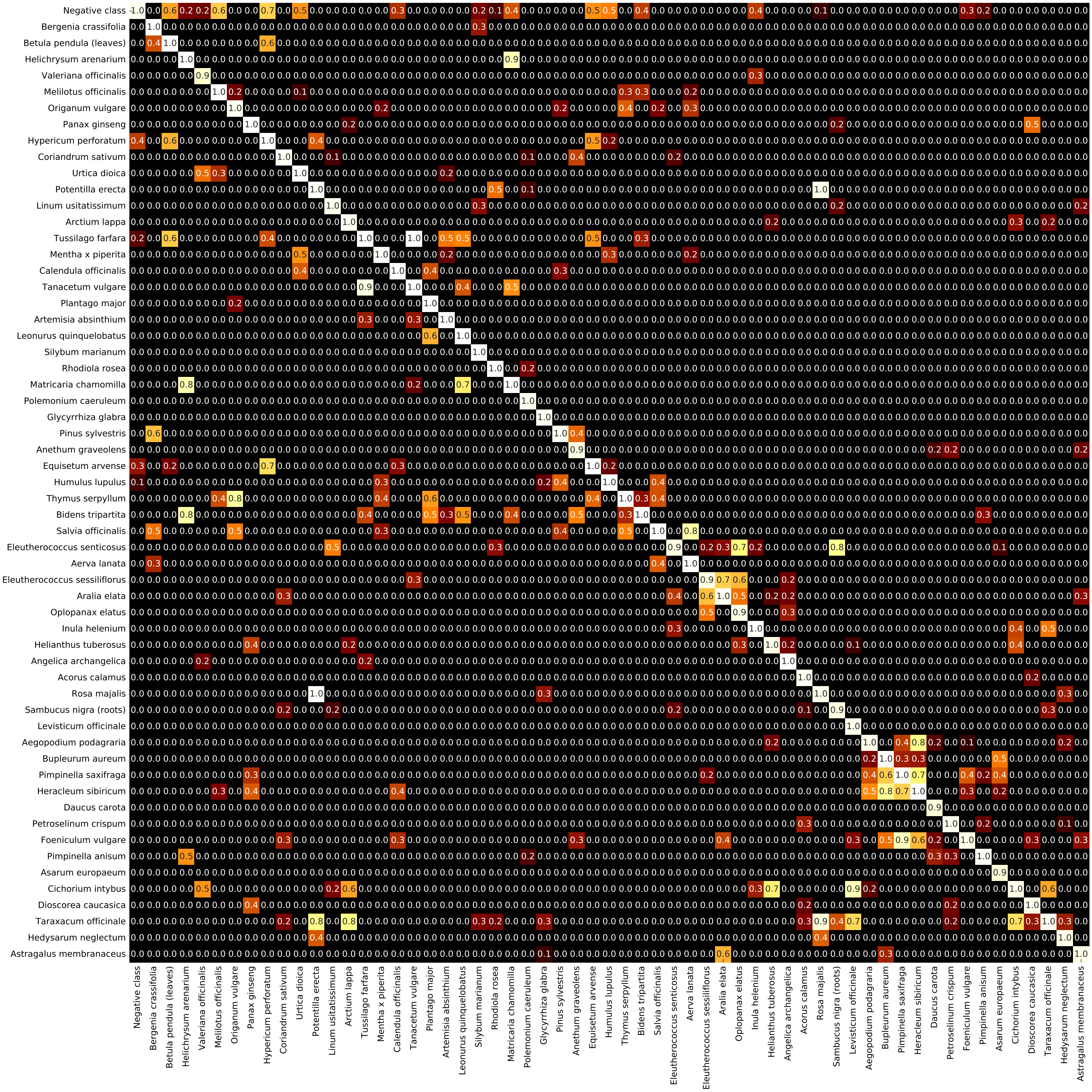

Figure S1.2(e): Sparse NTD classifier

3 Hierarchical clustering analysis (HCA)

HCA has been performed with hierarchical agglomerative clustering algorithm. Two variants of analysis have been performed: (1) clustering of mean samples for each of 76 classes (74 species); (2) clustering of all samples.

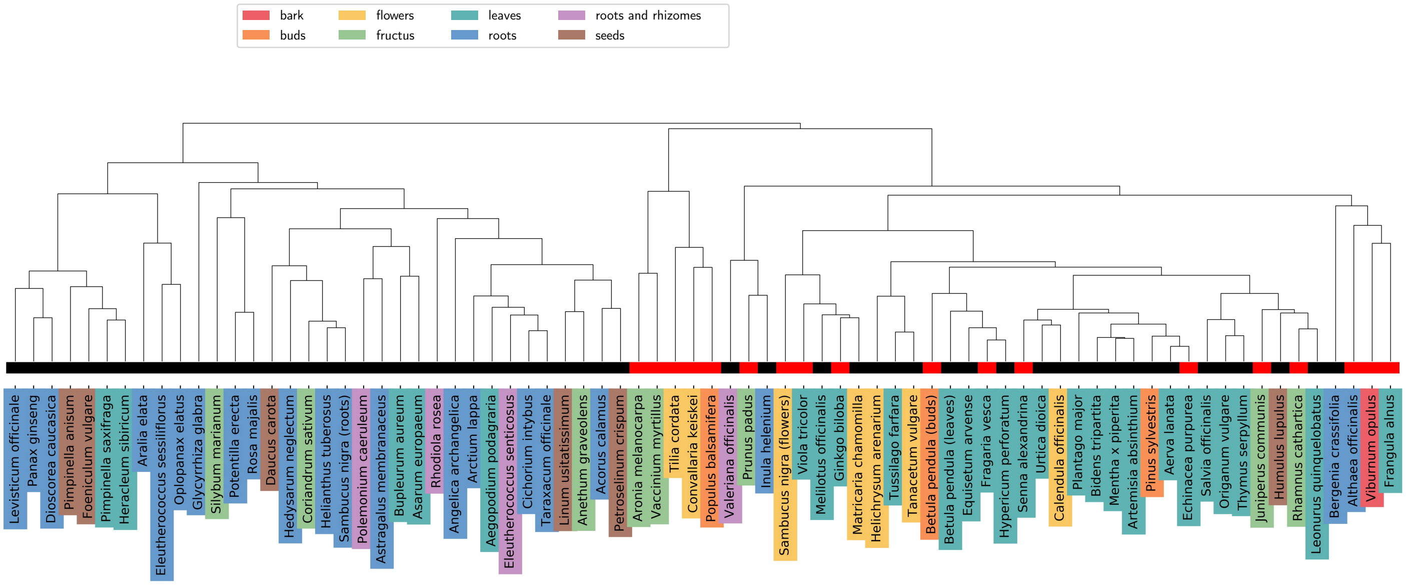

Figure S1.3(a): HCA of mean samples in the original feature space (linkage: complete, metric: correlation)

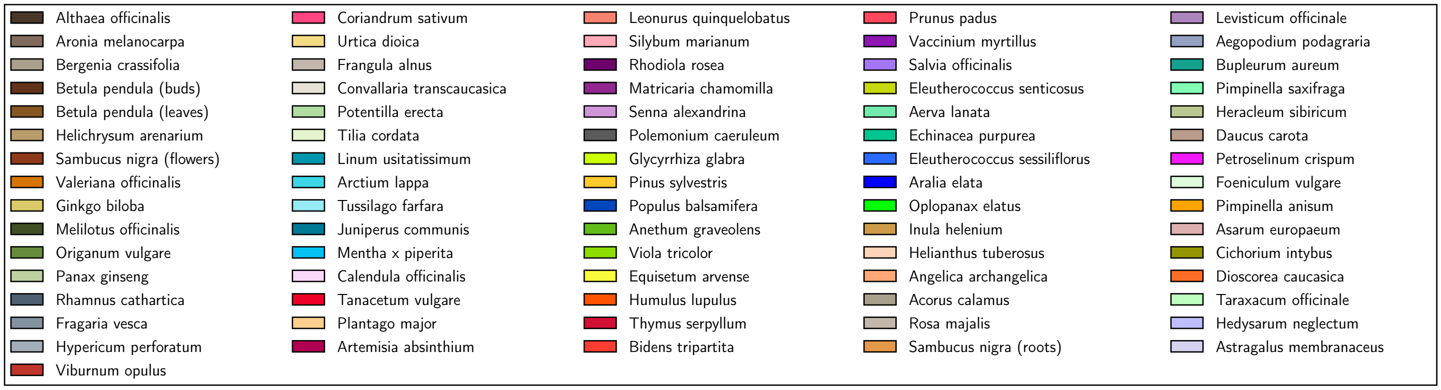

Figure S1.3(b): Colour codes for 76 classes

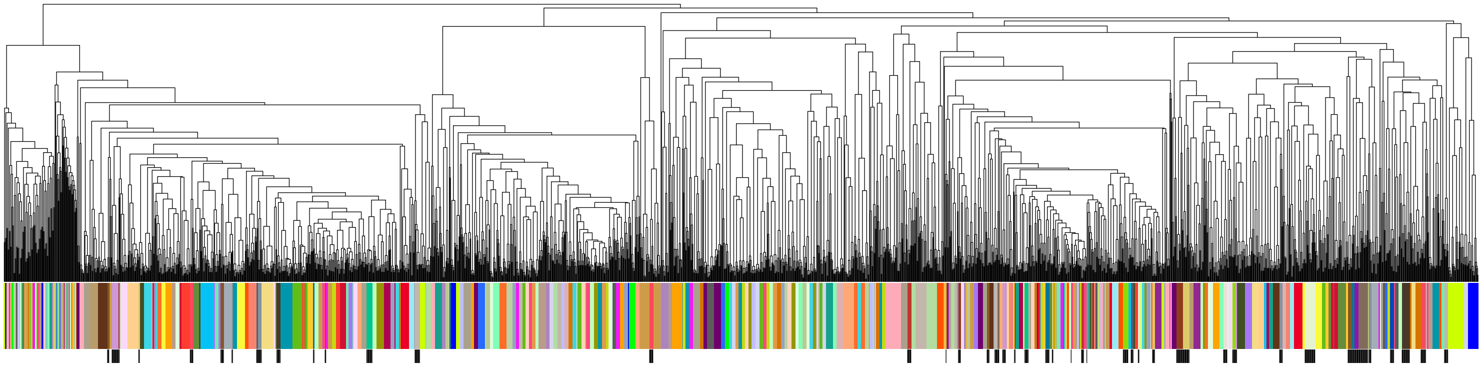

Figure S1.3(c): HCA of all samples in the original feature space (linkage: weighted, metric: euclidean); black vertical lines indicate samples from the negative class

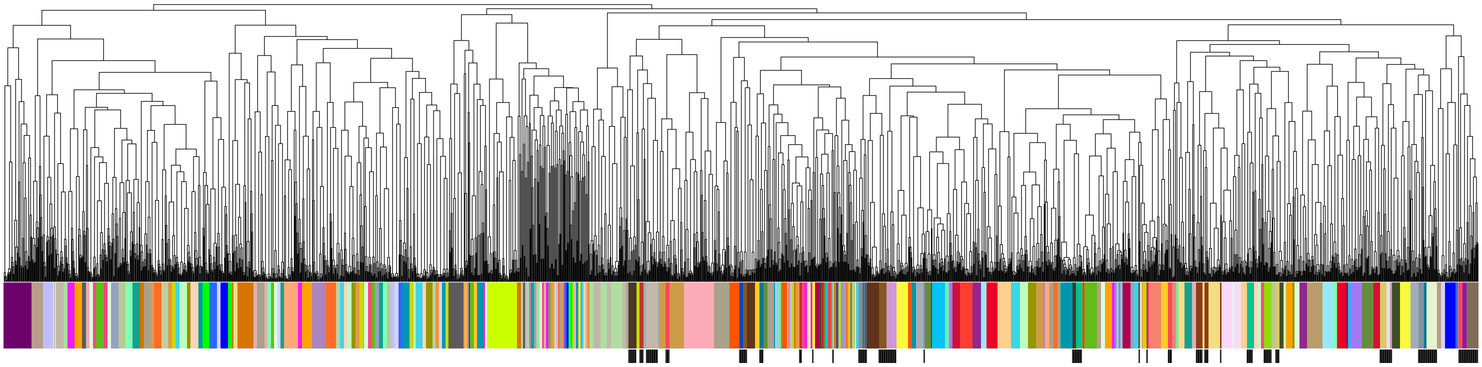

Figure S1.3(d): HCA of whole samples in the autoencoded feature space (linkage:weighted, metric: euclidean); black vertical lines indicate samples from the negative class

4 Sparse non-negative components

Components obtained after sparse non-negative matrix factorization (SNMF) and sparse non-negative Tucker decoposition (SNTD). On Figures S1.4(a), S1.4(b) first three components extracted by SNMF (and then splitted into positive and negative polarity parts) and SNTD algorithms are displayed. The following criteria was used to select three components:

$$\min_{i=\overline{1,r};i\notin\Omega} \left(\frac{1}{n_c-1}\sum_{j\neq l}|B[j,i]|\right) - \log(|A[1,i]|+\varepsilon), \tag{1}$$

where Ω is a set of already selected components, $B \in \mathbb{R}^{n_c - 1 \times r_l}$ - medians (taken by sample axis) of m/z-wise correlation matrix between components for current class l and samples drawn from other classes, and $A \in \mathbb{R}^{1 \times r_l}$ is a vector of sample-wise medians of correlation matrix between components of current class l and samples drawn from current class, n_c - summarized number of classes.

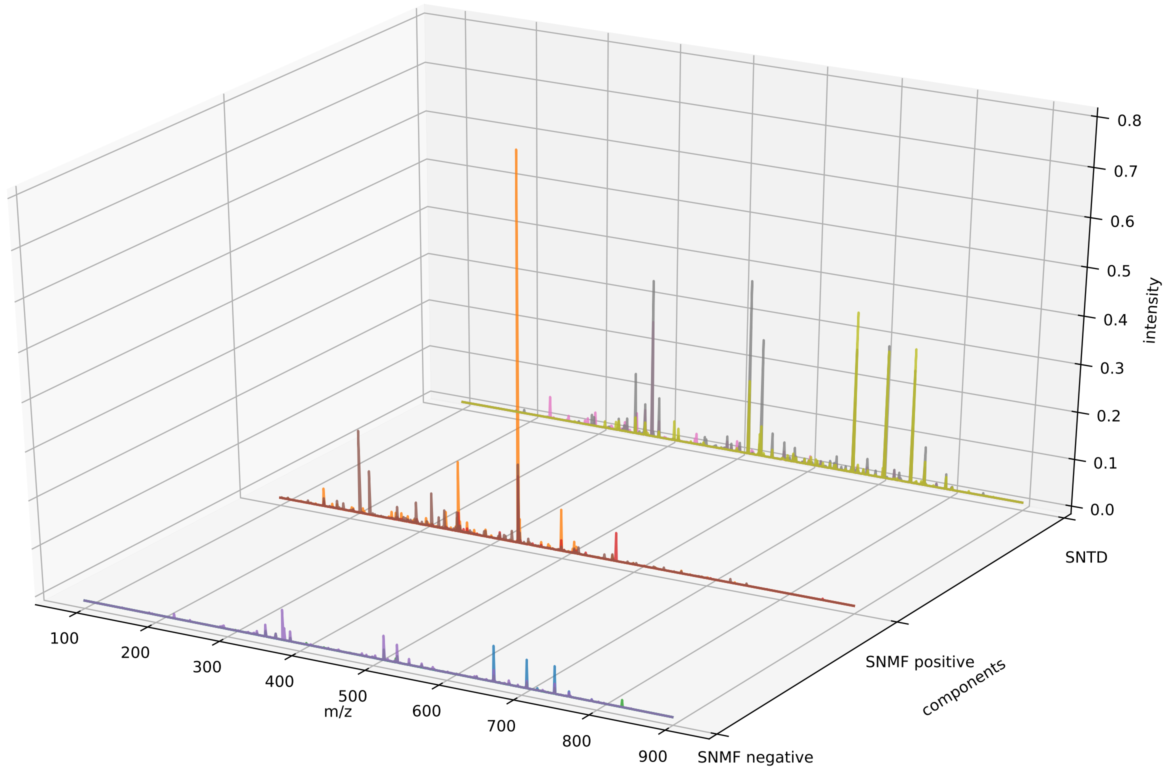

Figure S1.4(a): Aralia elata. Colours indicate separate components.

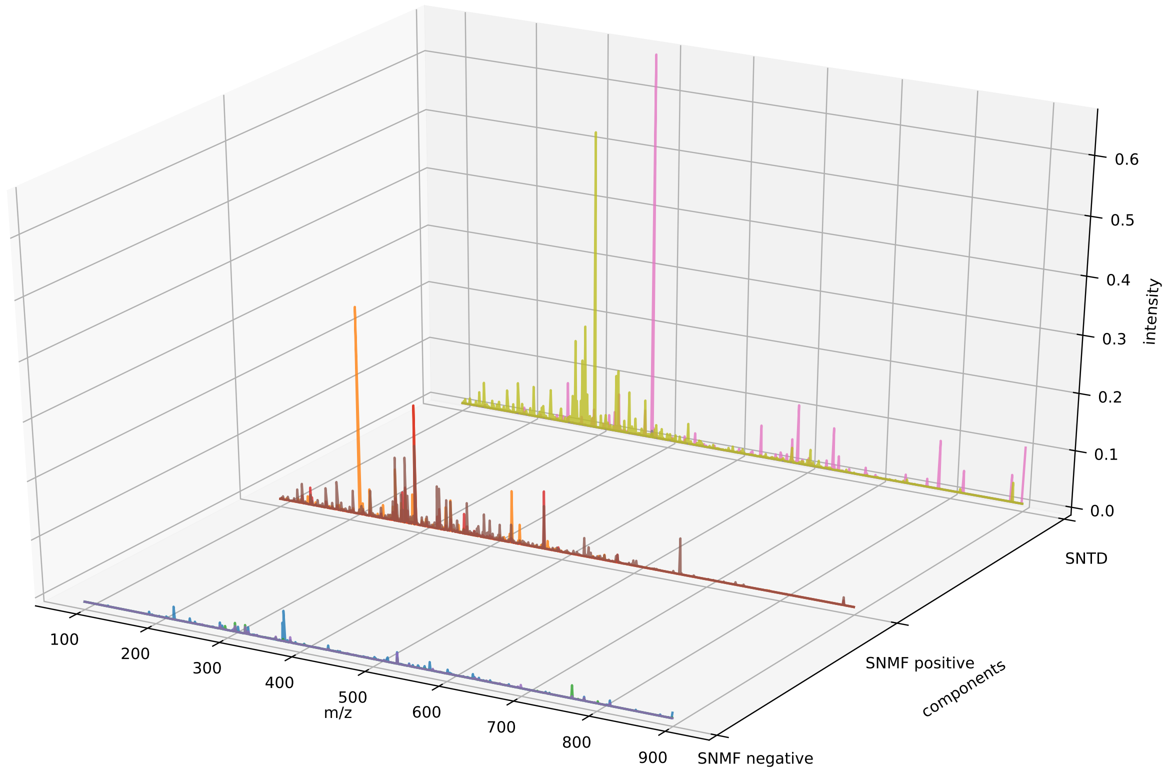

Figure S1.4(b): Dioscorea caucasica. Colours indicate separate components.

5 Autoencoder: structure, t-SNE plots and selection of last layer size

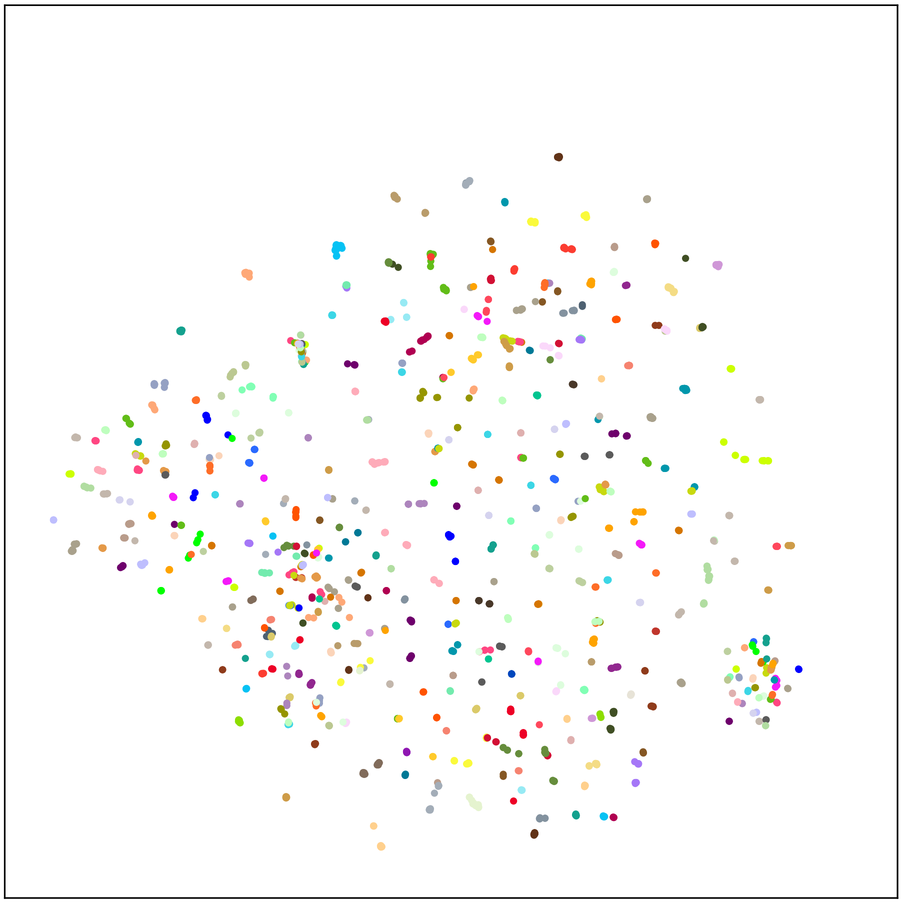

Figure S1.5(a): t-SNE plot for the main dataset with original feature space (1600 variables). Colour codes are the same as in Figure S1.3(b). Perplexity=10.

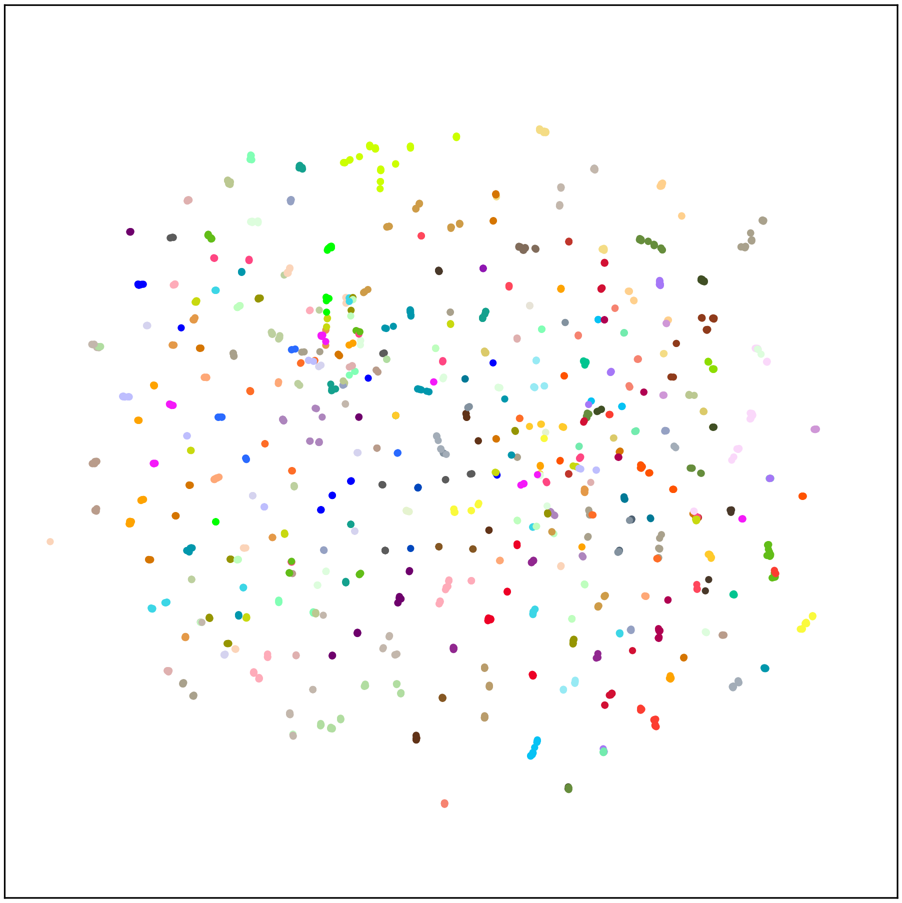

Figure S1.5(b): t-SNE plot for the main dataset with autoencoded feature space (25 variables). Colour codes are the same as in Figure S1.3(b). Perplexity=10.

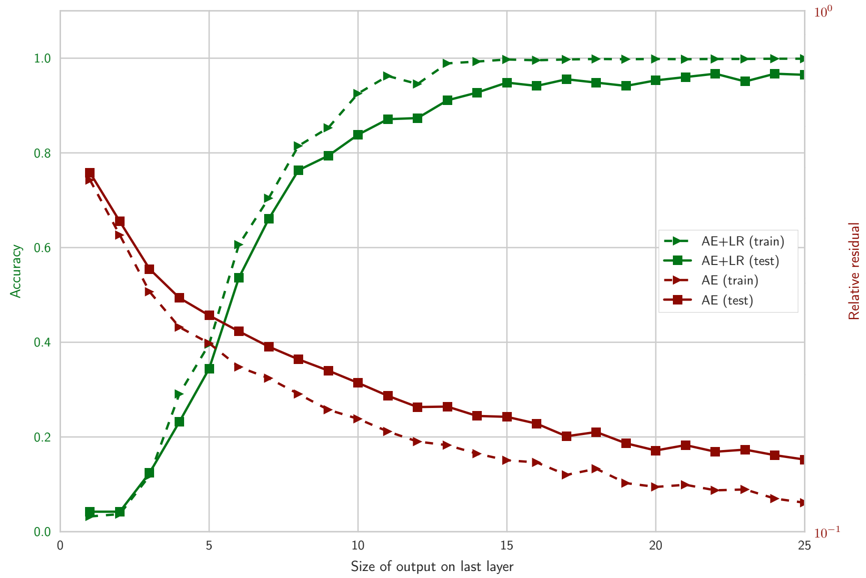

Figure S1.5(c): Accuracy on train/test1 parts of the dataset on 5-fold CV (green lines) and median of relative residual error among samples (red lines) depending on last layer size

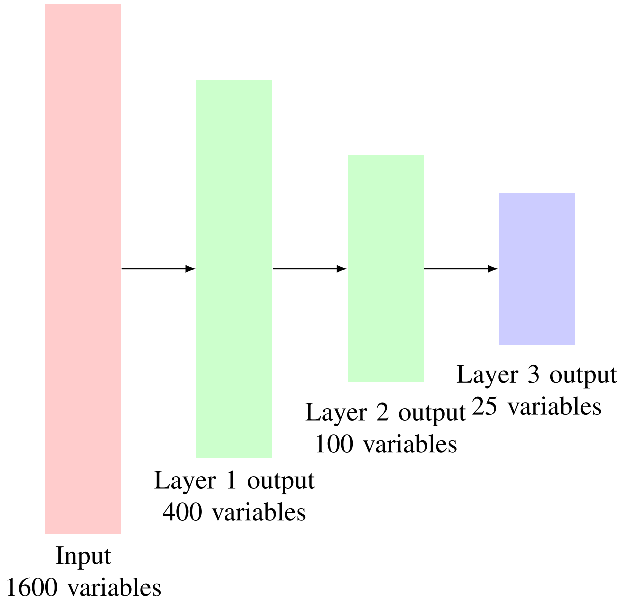

Figure S1.5(d): Final structure of autoencoder's encoding part

6 Dataset

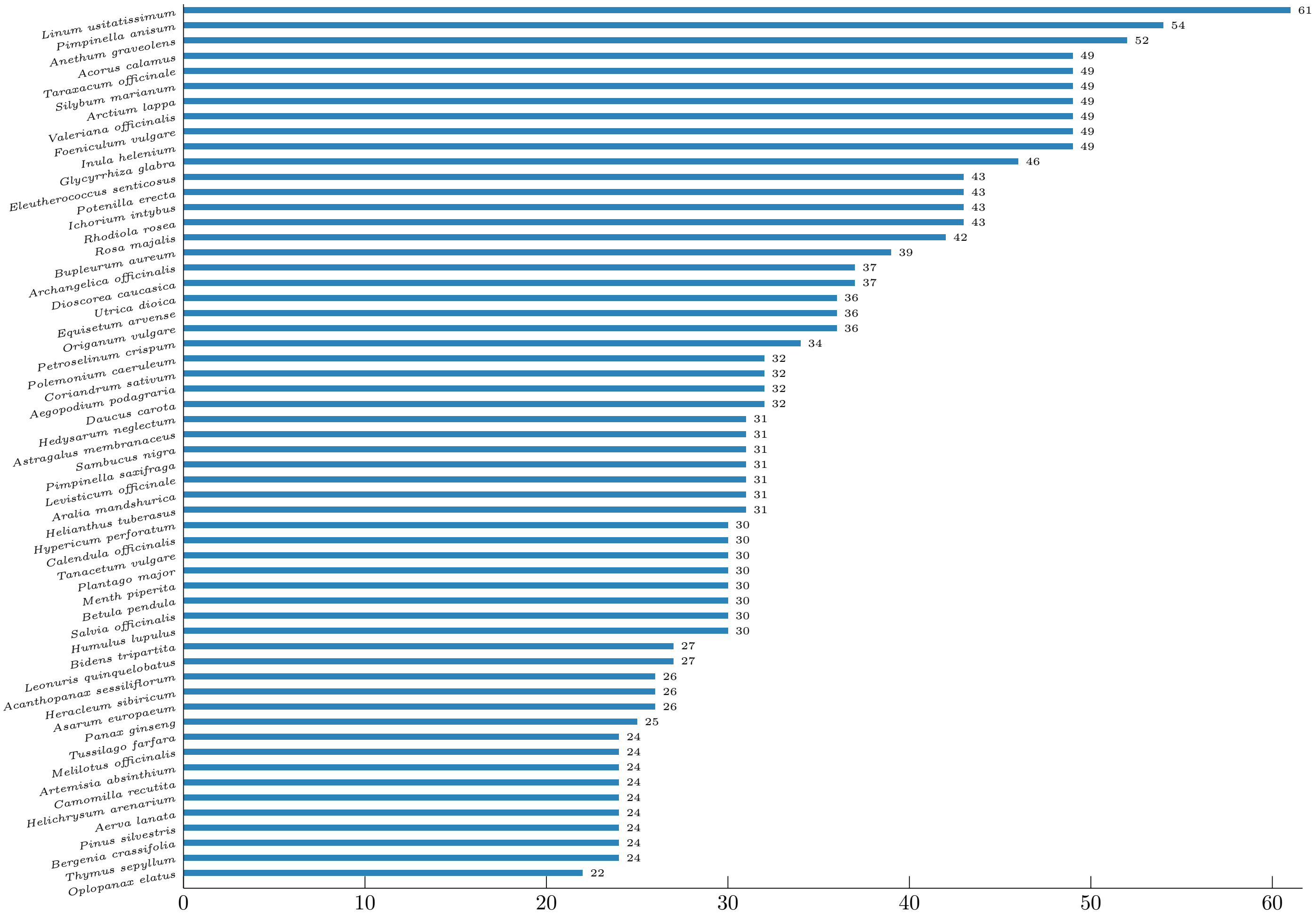

Figure S1.6(a): Dataset composition by plant species. Only 58 species with 20 or more available chromatograms are displayed.

Table S1.6(a): Plant species used in experiment and corresponding class labels. Corresponding file in computer readable format is available at Github repository `https://github.com/kharyuk/chemfin-plasp` as data/species.csv .

Class	Plant species	Organ	Class	Plant species	Organ
1	Althaea officinalis	roots	41	Glycyrrhiza glabra	roots
2	Aronia melanocarpa	fructus	42	Pinus sylvestris	buds
3	Bergenia crassifolia	roots	43	Populus balsamifera	buds
4	Betula pendula	leaves	44	Anethum graveolens	fructus
5	Betula pendula	buds	45	Viola tricolor	leaves
6	Helichrysum arenarium	flowers	46	Equisetum arvense	leaves
7	Sambucus nigra	flowers	47	Humulus lupulus	seeds
8	Valeriana officinalis	roots,	48	Thymus serpyllum	leaves
		rhizomes	49	Bidens tripartita	leaves
9	Ginkgo biloba	leaves	50	Prunus padus	fructus
10	Melilotus officinalis	leaves	51	Vaccinium myrtillus	fructus
11	Origanum vulgare	leaves	52	Salvia officinalis	leaves
12	Panax ginseng	roots	53	Eleutherococcus senticosus	roots,
13	Rhamnus cathartica	fructus			rhizomes
14	Fragaria vesca	leaves	54	Aerva lanata	leaves
15	Hypericum perforatum	leaves	55	Echinacea purpurea	leaves
16	Viburnum opulus	bark	56	Eleutherococcus sessiliflorus	roots
17	Coriandrum sativum	fructus	57	Aralia elata	roots
18	Urtica dioica	leaves	58	Oplopanax elatus	roots
20	Frangula alnus	leaves	59	Inula helenium	roots
23	Convallaria keiskei	flowers	60	Helianthus tuberosus	roots
24	Potentilla erecta	roots	61	Angelica archangelica	roots
25	Tilia cordata	flowers	62	Acorus calamus	roots
26	Linum usitatissimum	seeds	63	Rosa majalis	roots
27	Arctium lappa	roots	64	Sambucus nigra	roots
28	Tussilago farfara	leaves	65	Levisticum officinale	roots
29	Juniperus communis	fructus	66	Aegopodium podagraria	leaves
30	Mentha x piperita	leaves	67	Bupleurum aureum	leaves
31	Calendula officinalis	flowers	68	Pimpinella saxifraga	leaves
32	Tanacetum vulgare	flowers	69	Heracleum sibiricum	leaves
33	Plantago major	leaves	70	Daucus carota	seeds
34	Artemisia absinthium	leaves	71	Petroselinum crispum	seeds
35	Leonurus quinquelobatus	leaves	72	Foeniculum vulgare	seeds
36	Silybum marianum	fructus	73	Pimpinella anisum	seeds
37	Rhodiola rosea	roots,	75	Asarum europaeum	leaves
		rhizomes	76	Cichorium intybus	roots
38	Matricaria chamomilla	flowers	77	Dioscorea caucasica	roots
39	Senna alexandrina	leaves	78	Taraxacum officinale	roots
40	Polemonium caeruleum	roots,	79	Hedysarum neglectum	roots
		rhizomes	80	Astragalus membranaceus	roots

7 Confusion matrices for prediction of plant parts

In this section we provide confusion matrices measured on test1 part as medians of 5 times repeated 5-fold cross validation (25 runs in total). Columns: predicted labels; rows: true labels.

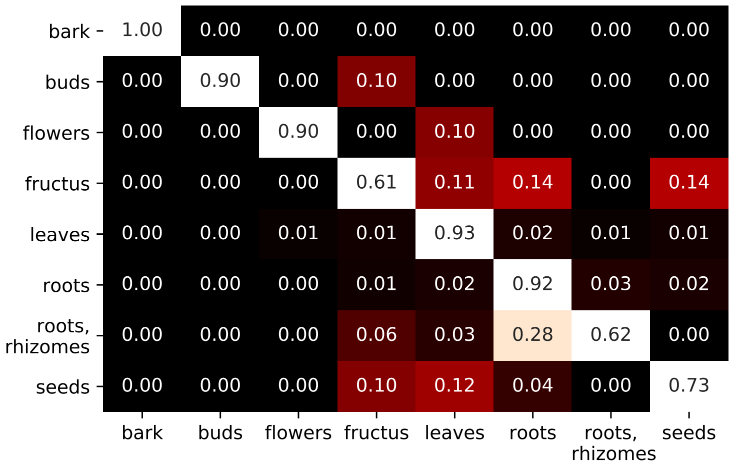

Figure S1.7(a): Median confusion matrix for Logistic Regression trained on autoencoded feature space.

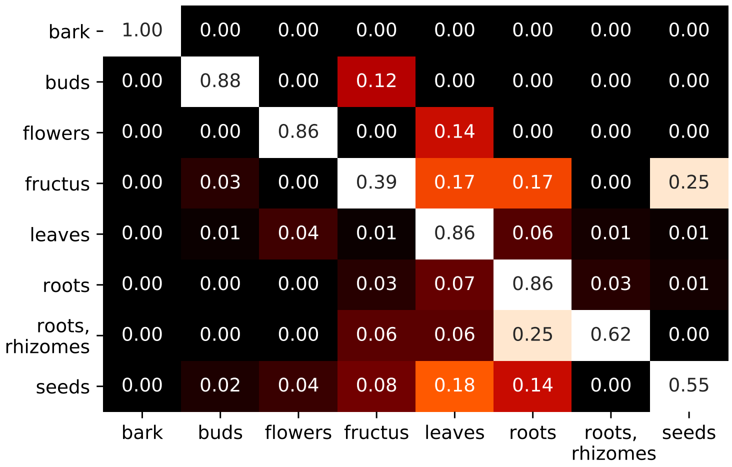

Figure S1.7(b): Median confusion matrix for Naive Bayes classifier trained on autoencoded feature space.

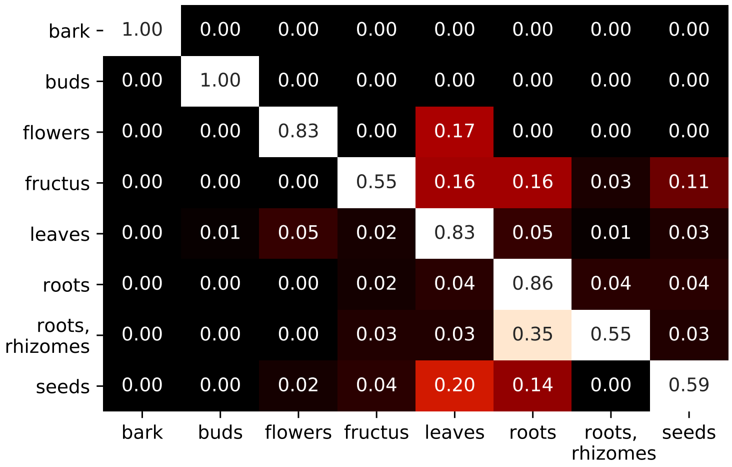

Figure S1.7(c): Median confusion matrix for Hybrid Bayesian Network trained on autoencoded feature space.

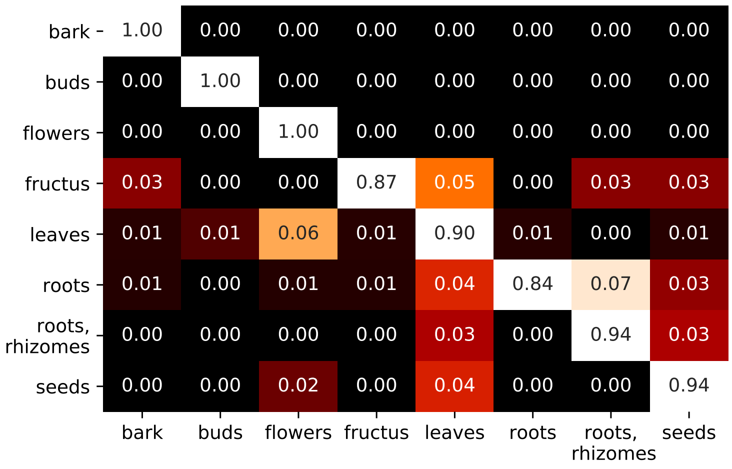

Figure S1.7(d): Median confusion matrix for classifier based on sparse non-negative Tucker decomposition.

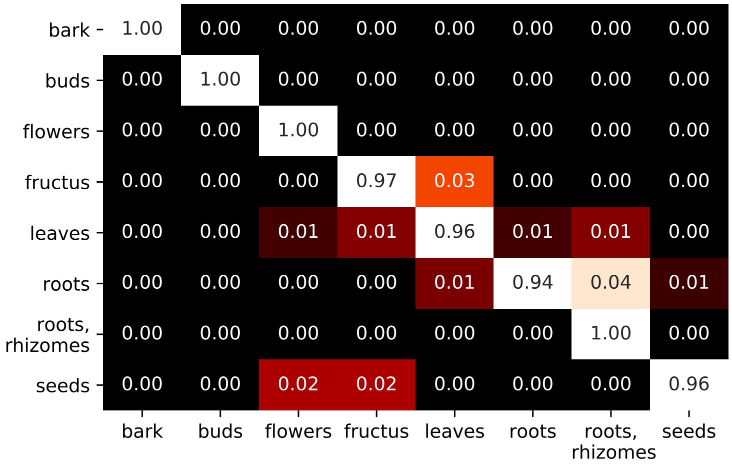

Figure S1.7(e): Median confusion matrix for classifier based on sparse non-negative matrix factorization.

8 Github repository structure.

Directory	Description
data	Directory with data (csv, sif, npz)
models	Directory for storing computed models
notebook	Computational experiments
results	Directory for storing computed results
src	Python sources

Table S1.8(a): Structure of chemfin-plasp repository (github, link: `https://github.com/kharyuk/chemfin-plasp`)
